# Glucosylceramide Associated with Gaucher Disease Forms Amyloid-like Twisted Ribbon Fibrils That Induce α-Synuclein Aggregation

**DOI:** 10.1021/acsnano.1c02957

**Published:** 2021-07-02

**Authors:** Ashim Paul, Guy Jacoby, Dana Laor Bar-Yosef, Roy Beck, Ehud Gazit, Daniel Segal

**Affiliations:** † Department of Molecular Microbiology and Biotechnology, Shmunis School of Biomedicine and Cancer Research, Tel Aviv University, Ramat Aviv, Tel Aviv 6997801, Israel; ‡ The Raymond and Beverly Sackler School of Physics and Astronomy, The Center for Nanoscience and Nanotechnology, and the Center for Physics and Chemistry of Living Systems, Tel Aviv University, Tel Aviv 69978, Israel; § Department of Materials Science and Engineering, Iby and Aladar Fleischman Faculty of Engineering, Tel Aviv University, Tel Aviv 69978, Israel; ∥ Sagol Interdisciplinary School of Neuroscience, Tel Aviv University, Ramat Aviv, Tel Aviv 6997801, Israel

**Keywords:** Gaucher disease, Parkinson’s disease, glucosylceramide, amyloid-like aggregation, α-synuclein, cross-seeding

## Abstract

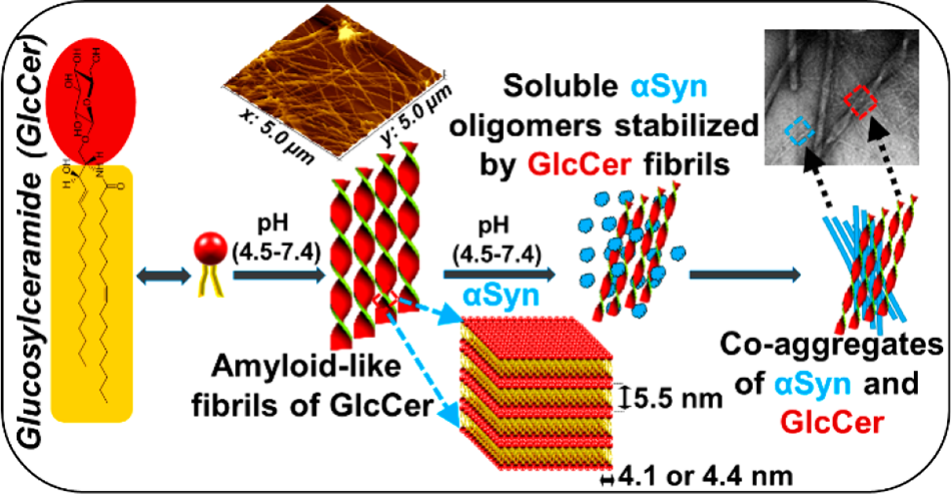

A major risk factor for Gaucher’s disease is loss of function mutations in the *GBA1* gene that encodes lysosomal β-glucocerebrosidase, resulting in accumulation of glucosylceramide (GlcCer), a key lysosomal sphingolipid. *GBA1* mutations also enhance the risk for Parkinson’s disease, whose hallmark is the aggregation of α-synuclein (αSyn). However, the role of accumulated GlcCer in αSyn aggregation is not completely understood. Using various biophysical assays, we demonstrate that GlcCer self-assembles to form amyloid-like fibrillar aggregates *in vitro*. The GlcCer assemblies are stable in aqueous media of different pH and exhibit a twisted ribbon-like structure. Near lysosomal pH GlcCer aggregates induced αSyn aggregation and stabilized its nascent oligomers. We found that several *bona fide* inhibitors of proteinaceous amyloids effectively inhibited aggregation of GlcCer. This study contributes to the growing evidence of cross-talk between proteinaceous amyloids and amyloid-like aggregates of metabolites accumulated in diseases and suggests these aggregates as therapeutic targets.

Protein aggregation and amyloid formation account for the onset of various human disorders, including neurodegenerative diseases such as Alzheimer’s disease (AD) and Parkinson’s diseases (PD).^1,2^ In the amyloid state, the self-assembled protein is arranged in a highly organized and stable fibrillar structure, which is rich in β-sheet conformation.^2,3^ The amyloid assemblies exist in various isoforms (oligomers, protofibrils, and mature fibrils), and their deposition in the extra or intracellular spaces is the primary cause of these diseases.^1−3^ The mechanism of amyloid formation, specifically at the early stage of aggregation, remains elusive.

Recently, various metabolites, which accumulate in a range of human diseases, collectively termed inborn errors of metabolism (IEM), including amino acids and nucleobases, were found to form amyloid-like aggregates.^4−6^ For example, phenylalanine (Phe), accumulated in phenylketonuria (PKU) was recently reported to form well-ordered fibrillar structures *in vitro*, similar to those of classical protein amyloids. Notably, Phe aggregates are present in the hippocampus of model mice as well as in the parietal cortex of brain tissue from PKU patients.^5^ Similarly, other metabolites, including homogentisic acid, adenine, orotic acid, cystine, homocysteine, tyrosine, tryptophan, glycine, histidine, quinolinic acid, and uracil, were found to form *in vitro* ordered structures that greatly resemble assemblies of proteinaceous amyloids and mostly are associated with various human diseases.^4,6−9^ Among these, in addition to Phe, homogentisic acid, and adenine, were found to form amyloid-like structures *in vivo.*(5,8,10,11) An intriguing effect of these metabolite aggregates is their ability to promote aggregation of various proteins, including α-synuclein (αSyn), serum amyloid A, and Aβ42, under physiological conditions, very similar to the cross-seeding of aggregation of proteinaceous amyloids proteins by heterologous amyloidogenic proteins.^8−10,12^ Mitigation of amyloid-like metabolite assemblies in IEMs is an unmet need.

Impairment of sphingolipids metabolism is implicated in various IEMs.^13,14^ Gaucher disease (GD) is an autosomal recessive IEM caused by loss of function mutations in the gene GTP-binding protein type A1 (*GBA1*) that encodes lysosomal hydrolase β-glucocerebrosidase 1 (GCase1) in the lysosomes of the mononuclear phagocytes system.^15,16^ A major function of GCase1 is to cleave the β-glucosidic linkage of glucocerebroside lipids, for instance, the hydrolysis of glucosylceramide (GlcCer) into glucose and ceramide.^17^ In GD, the deficiency or mutation in GCase1 lead to aberrant accumulation of GlcCer primarily within the lysosome.^16,18^ The most frequent GD symptoms are splenomegaly, hepatomegaly, cytopenia, and potentially severe involvement of the bone.^16,19^ Neurological manifestation may occur in more severe types of the disease.^19,20^ GlcCer, the simplest among glycosphingolipids (GSLs), is synthesized at the cytosolic leaflet of the Golgi and is translocated to the plasma membrane and the membrane of cellular organelles.^17,21^ GlcCer participates in various fundamental processes and cell-specific functions in mammals, including being a precursor for the highly polymorphic set of complex GSLs, protein sorting, signaling, and membrane deformation.^21,22^ The loss of function of GCase1 in GD results in the accumulation of GlcCer and downstream bioactive lipids, for instance, glucosylsphingosine (GlcSph), due to alterations in glycosphingolipid homeostasis.^23^

Interestingly, GD patients were estimated to have a 20- to 30-fold increased risk for developing PD.^18,24,25^ Loss of GCase1 activity is also found in sporadic PD brains. The two most frequent *GBA1* mutations associated with PD (N370S and L444P) are found in 17–31% of all PD patients in European Ashkenazi Jewish populations and 3% of non-Ashkenazi populations.^26^ Aggregation of αSyn into amyloid fibrils is a hallmark PD.^1^ However, the underlining cause for the correlation between GD and PD remains to be established.^27^ It has been proposed, for example, that the accumulation of the GD-substrates alters the homeostasis of αSyn in the lysosomes, that the mutant GCase1 causes saturation of the ubiquitin-proteasome pathway, thereby reducing its activity of degradation of αSyn, or that loss of GCase1 function impairs activity and structure of the mitochondria, a process that can aggravate accumulation of αSyn.^28^ Recent *in vitro* and *in cell* experiments revealed that incubation of αSyn with liposomes containing either GlcCer, GlcSph, sphingosine, or sphingosine-1-phosphate leads to aggregation of αSyn, and the resulting oligomers were capable of cross-seeding endogenous αSyn in cultured human neurons.^13,29,30^

Current therapeutic approaches for GD focus primarily on overcoming the metabolic block caused by the mutations in the *GBA1* gene and restoring the biosynthetic pathway.^31^ Replacement of the defective GCase1 enzyme by routine infusion of recombinant normal GCase1 has been very successful, especially for type 1 GD, albeit highly costly. Such enzyme replacement lessens the visceral, hematological, and sometimes skeletal pathologies of GD; however, since it cannot cross the blood–brain barrier, it does not affect neurological involvement of GD.^31^ Pharmacological chaperones to mitigate misfolding of mutant GCase1 are also being developed as an alternative treatment approach for GD, for example, Ambroxol.^32^ In another recent therapeutic approach for GD, termed ‘substrate reduction therapy’, rather than enhancing the deficient enzyme levels, the drugs target an earlier step in the metabolic pathway, leading to inhibition of glucocerebroside production, the lipid that is not being efficiently cleaved by the mutant GCase1.^33^ However, to the best of our knowledge, no therapeutic strategies currently aim at directly targeting the accumulated GlcCer. Recently some small molecules, including doxycycline, polyphenols, and quinones, which are effective inhibitors of aggregation of proteinaceous amyloids, were found to be efficacious in inhibiting amyloid-like self-assembly of certain accumulated metabolites.^11,34−36^

Here, using various *in vitro* studies, including Thioflavin T (ThT) binding, turbidity assay, electron microscopy, and fluorescence microscopy, we demonstrate that the onset of accumulation of GlcCer, characteristic of GD, proceeds through the formation of amyloid-like aggregates. X-ray scattering analysis of the GlcCer aggregates enabled us to propose a plausible model that satisfies their twisted-ribbon-like structure. We observed an inducing effect of GlcCer self-assemblies on the aggregation of αSyn, suggesting a plausible mechanism linking GD and PD pathologies. Finally, we demonstrate that several small molecules, including allantoin, rosmarinic acid (RA), epigallocatechin gallate (EGCG), and naphthoquinone-tryptophan (NQTrp), known to inhibit aggregation of proteinaceous amyloids, efficiently modulate GlcCer aggregation *in vitro*.

## Results and Discussion

GlcCer (Figure S1) powder was dissolved in 100% DMSO to make a stock solution (100 mM), which we refer to as monomers. It was diluted with PBS (pH 7.4) to make various concentrations and was immediately used. When GlcCer (100 μM) was incubated in PBS buffer for 10 h, it formed a fibrillary-like morphology when viewed under transmission electron microscopy (TEM) and atomic force microscopy (AFM) (Figure S2). The morphology of the GlcCer aggregates looks very similar to that of *bona fide* proteinogenic amyloid fibrils.^1,37^ The critical concentration for fibril formation by GlcCer was further evaluated using TEM, and we observed that upon dilution from 50 μM to 100 nM, the fibrillary morphology remained the same and no fibrils were noticed below 100 nM concentration (Figure S3). To examine whether these fibrils exhibit amyloid-like properties, we performed several biophysical assays, which are commonly used to characterize proteinaceous amyloid structures.^1,37−39^

### Accumulated GlcCer Forms Amyloid-like Aggregates

**Figure 1:**
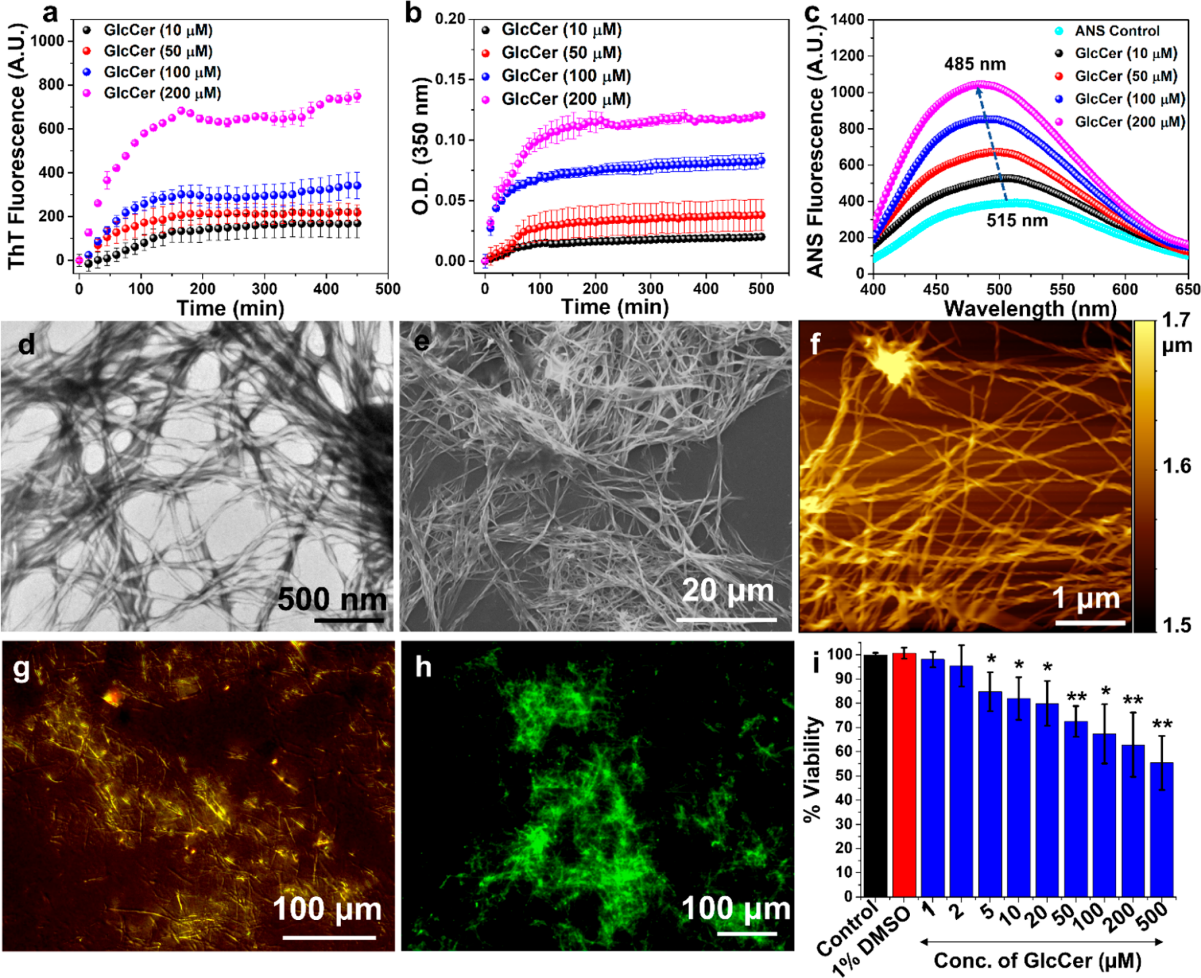
Amyloid-like characteristics of GlcCer assemblies. (a) Time-dependent ThT binding kinetics, (b) Turbidity assay, (c) ANS-binding assay for the aggregation of GlcCer at different concentrations (10–200 μM). (d) TEM, (e) SEM, and (f) AFM images of GlcCer assemblies (200 μM). (g) Congo red stained birefringence of GlcCer assemblies. (h) Fluorescence imaging upon staining with ThT dye. (i) Cytotoxicity of GlcCer assemblies toward SH-SY5Y cells. Cells were incubated with preaggregated GlcCer (1–500 μM) for 24 h, and % viability was measured by XTT assay. 1% DMSO was used as a vehicle to solubilize GlcCer. Untreated cells were used as control and set to 100% viability. Results are an average of 6 replicates (*n* = 6, ± SD) and are expressed as a percentage of control cells. Significance: *p < 0.05, **p < 0.005, and ***p < 0.001. All the experiments were repeated at least 3 times with similar observation.

ThT is an amyloid reporter dye commonly used to monitor the kinetics of protein aggregation and quantification of amyloid formation.^39^ GlcCer was incubated at different concentrations (10–200 μM) in PBS at pH 7.4 and 37 °C, and its aggregation was monitored using a ThT fluorescence assay (Figure 1a). ThT fluorescence increased with time (Figure 1a), suggesting that GlcCer binds with ThT and possibly aggregates during that time course. With increasing concentrations of GlcCer, the rate of aggregation and the extent of ThT fluorescence were enhanced, indicating that GlcCer aggregates and forms amyloid-like structures in a concentration-dependent manner. The kinetics of GlcCer aggregation was further examined by turbidity assay and observed that it slowly formed a turbid solution, absorption monitored at 350 nm (Figures 1b and S4), very similar to reported amyloidogenic proteins.^40,41^ At a higher (200 μM) concentration of GlcCer, the solution became turbid faster than at lower concentrations (Figure 1b). To validate the amyloidogenic nature of the GlcCer aggregates, we used 1-anilinonaphthalene-8-sulfonic acid (ANS), commonly used to identify the hydrophobic patches of amyloids by a blue shift of its emission maximum (from ∼530 nm to ∼475 nm) upon binding to amyloids.^42^ A sharp blue shift (to ∼485 nm) with enhanced intensity was observed when ANS bound to different concentrations of GlcCer, whereas ANS alone (control) exhibited emission at ∼515 nm (Figure 1c). This indicates an amyloidogenic nature of the GlcCer aggregates and suggests that the accumulated ceramide’s hydrophobic tail interacted with ANS.

The morphology of the GlcCer assemblies at the end point of the ThT binding assay was examined using TEM and exhibited a fibrillar network (Figures 1d and S5), very similar to fibrils reported for proteinaceous amyloids and amyloid-like assemblies of accumulated metabolites.^4,6,43^ The density of the fibrillar network was higher as the concentration of GlcCer increased (Figure S5). We monitored, using TEM, the formation of GlcCer (100 μM) fibrils at different time points immediately after dissolution in PBS buffer from the stock solution (10 mM in DMSO). GlcCer did not show any fibrillary structure in DMSO (which we considered as monomers), and immediately after dissolution in PBS (at time 0), small aggregates were noticed and clear fibrils were evident upon further incubation (Figure S6). These fibrils remained very stable even after incubation for 60 days (Figure S7). We also examined the thermal stability of the GlcCer fibrils using TEM and observed that the fibrillar morphology remained stable from 25 to 80 °C (Figure S8). Scanning electron microscopy (SEM, Figure 1e) and AFM (Figure 1f) also revealed a fibrillar network of the GlcCer assemblies (100 μM), suggesting amyloid-like aggregate formation. Staining of the assemblies with Congo red, commonly used for amyloid characterization,^39,44^ exhibited golden-green birefringence, under cross-polarized light, typical of amyloid assemblies (Figure 1g). The GlcCer aggregates were found to remain stable in 80% aqueous EtOH, which was used for preparation of the Congo red solution (Figure S9). Using fluorescence microscopy, we also observed that the GlcCer assemblies were stained with ThT (Figure 1h), suggesting the formation of amyloid-like fibrils.

Amyloids produced by proteins or metabolites can be toxic to cultured neuronal cells.^1,8,12^ We therefore examined the cytotoxicity of the GlcCer assemblies toward human neuroblastoma SH-SY5Y cells using a XTT viability assay. With increased concentrations of GlcCer, the viability of the cells was reduced compared to the untreated cells (Figure 1i), suggesting cytotoxicity by the preaggregated GlcCer. Collectively, the results of these assays support the notion that GlcCer forms cytotoxic amyloid-like assemblies.

### Structural Arrangement of GlcCer Assemblies and Predicted Model

Accumulation of GlcCer in GD occurs in the lysosome which is acidic (pH 4.5–5), whereas its amyloid-like characteristics described above were recorded at pH 7.4 and 37 °C. Therefore, we examined the effect of pH on the formation of amyloid-like fibrils and the structural arrangement of GlcCer assemblies. To that end, ThT assay, turbidity assay, and TEM analysis of GlcCer aggregation were performed at different pHs (4.5 to 7.4), using four different pH media, 100 mM acetate buffer (AB, pH 4.5), double-distilled or deionized water (DDW, molecular biology grade water, pH ∼ 6), and 100 mM phosphate buffer (pH 6.5 and 7.4).

**Figure 2:**
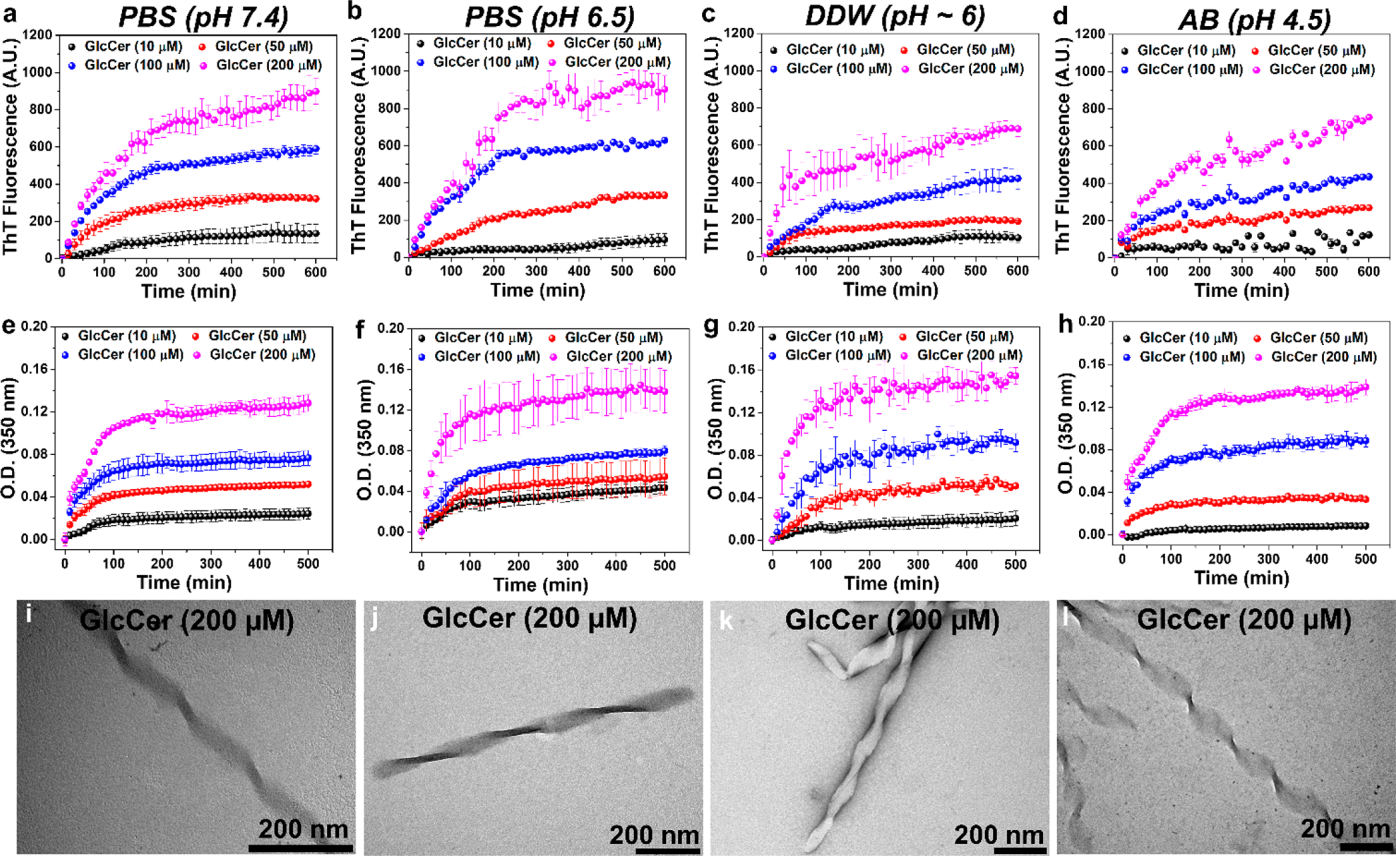
Effect of pH on GlcCer self-assembly. Time-dependent ThT kinetics for the aggregation of GlcCer at different concentrations in: (a) PBS pH 7.4, (b) PBS pH 6.5, (c) DDW pH ∼ 6, and (d) AB pH 4.5. Turbidity assay for the aggregation of GlcCer at different concentrations in: (e) PBS pH 7.4, (f) PBS pH 6.5, (g) DDW pH ∼ 6, and (h) AB pH 4.5. TEM images of GlcCer aggregate at 200 μM in: (i) PBS pH 7.4, (j) PBS pH 6.5, (k) DDW pH ∼ 6, and (l) AB pH 4.5. All experiments were repeated at least 3 times with similar observation.

As evident by the ThT and turbidity assays, in DDW, AB, or pH 6.5 PBS, GlcCer aggregated similarly to that in PBS at pH 7.4 (Figures 1a,b, 2a–h, and S10). Further, TEM analysis of GlcCer assemblies at different concentrations showed a similar amyloid-like fibrillar network at all tested pHs (Figure S11). These results suggest that the self-assembly of GlcCer depends mainly on the aqueous environment irrespective of the pH. Therefore, the structural characterization of GlcCer fibrils was further investigated in DDW (pH ∼ 6).

**Figure 3:**
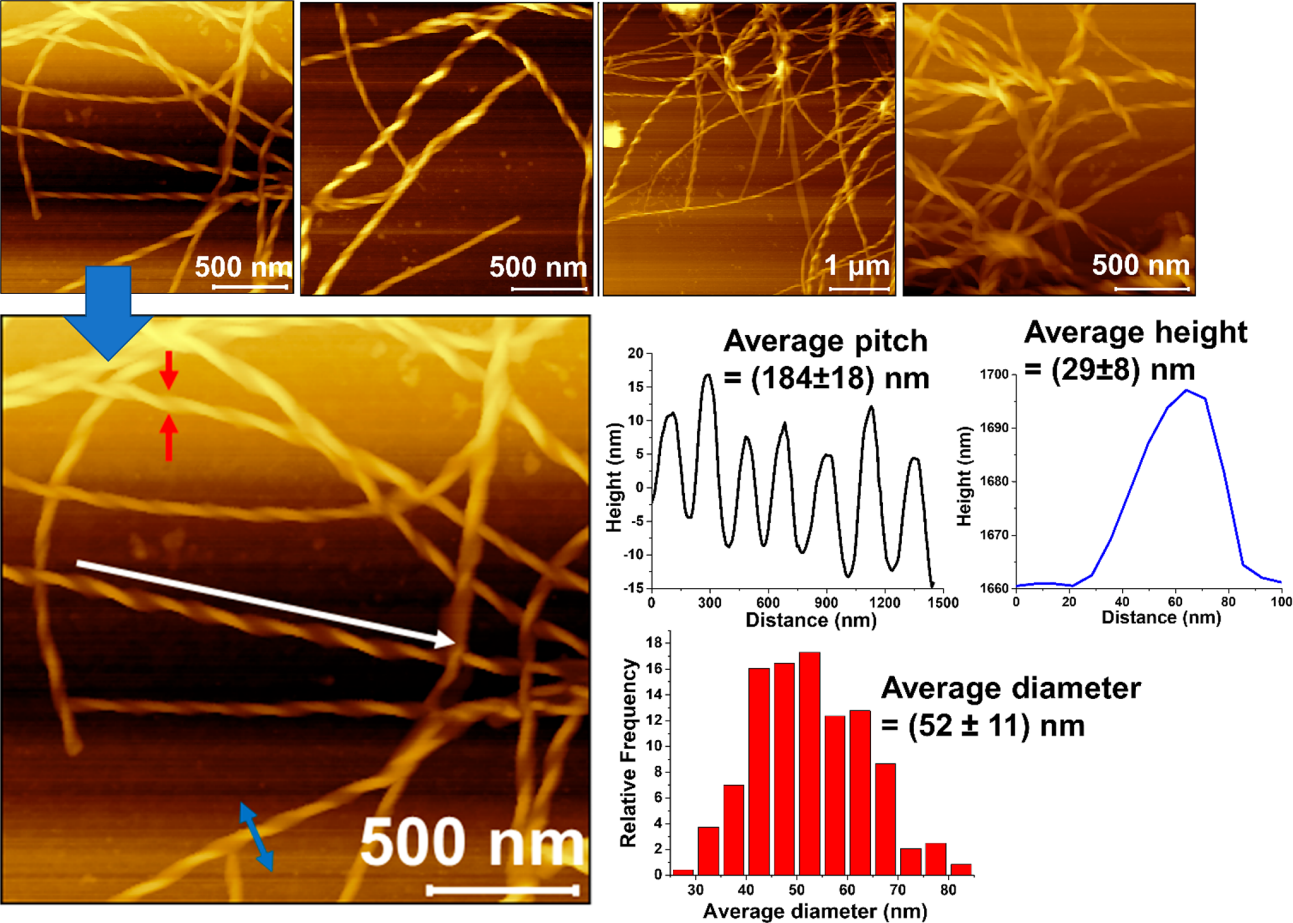
AFM images of GlcCer fibrils (200 μM). The average pitch/twist, width (fibril diameter), and the GlcCer fibrils’ height were calculated from three different AFM images, and their averages have been plotted. For calculating the average twist, 10 different fibrils were selected. For the average width and height calculations, 50 different regions have been selected from each AFM image.

A closer examination of the amyloid-like fibrils of GlcCer under TEM revealed a twisted-ribbon-like structure (Figure 2i–l). Calculated from AFM analysis, the dimensions of the twisted-ribbon-like GlcCer fibrils (100 μM) were an average twist (pitch) of 184 ± 18 nm, width (diameter) of 52 ± 11 nm, and height of 29 ± 8 nm (Figures 3 and S12). These parameters resemble the previously reported twisted helical GlcCer fibrils obtained from GD patients’ brain^45^ and similar to those observed for *in vitro* generated galactosylceramide (GalCer) fibrils.^46,47^

**Figure 4:**
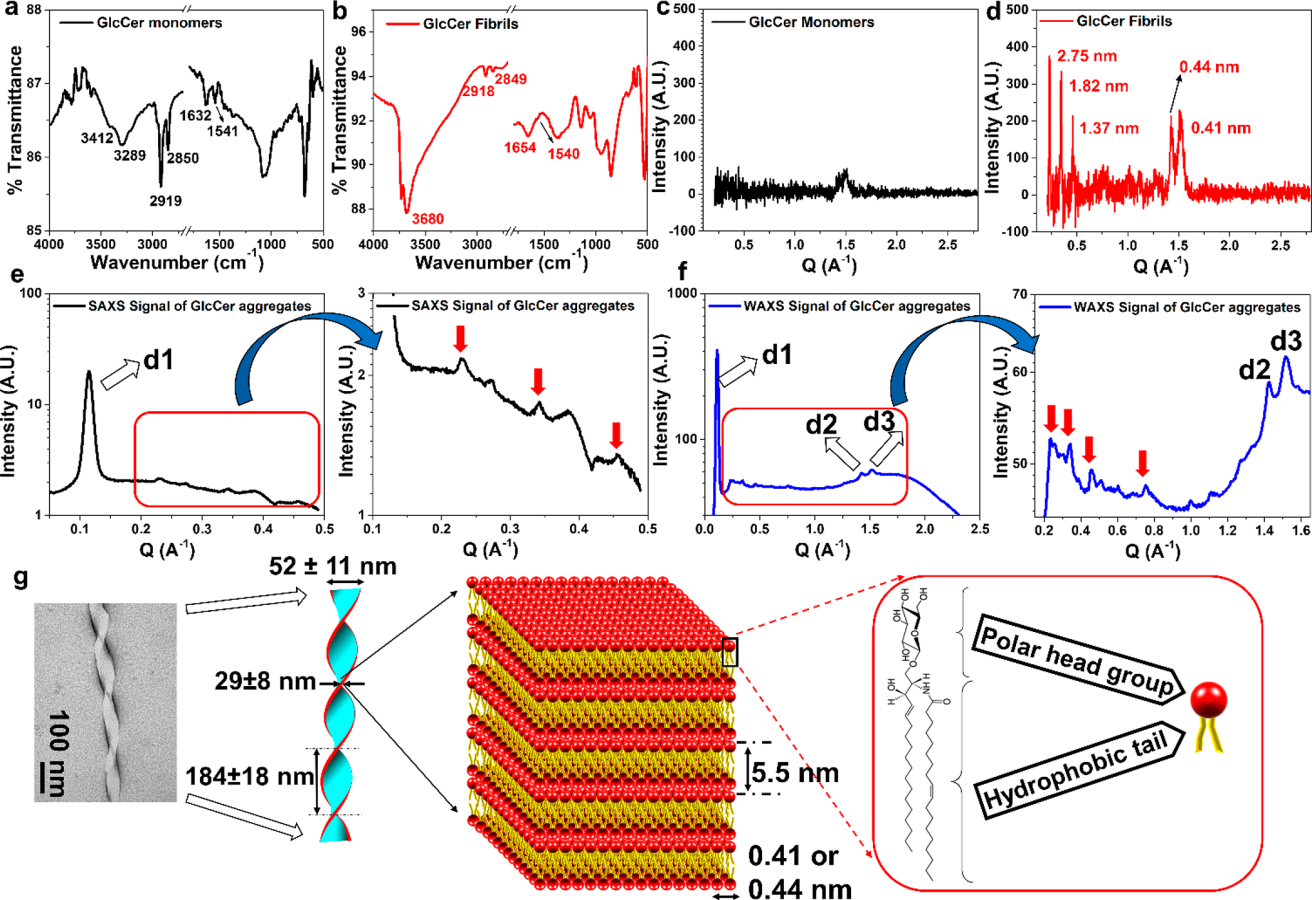
Structural parameters of GlcCer fibrils. FITR spectra of (a) GlcCer monomer and (b) fibrils. PXRD of the (c) GlcCer monomer and (d) fibrils. (e) SAXS pattern of the GlcCer fibrils and the corresponding X-ray harmonics are marked (red arrow). (f) WAXS pattern of the GlcCer fibrils and the corresponding X-ray harmonics are marked (red arrow). All of the reflections are labeled. (g) Plausible model of the twisted ribbon-like GlcCer fibrils predicted based on the parameters calculated from AFM images and X-ray scattering analysis.

To get additional structural insights and fit a twisted-ribbon model, we utilized TEM, AFM, Fourier-transform infrared (FTIR), powder X-ray diffraction (PXRD), and small- and wide-angle X-ray scattering (SAXS and WAXS) analyses. FTIR spectroscopy provides information on all functional groups present in a molecule^48,49^ and has been extensively used to understand the molecular interaction between lipids, including sphingolipids, within bilayers in aqueous media.^48^ FTIR analysis revealed that the monomeric GlcCer exhibited two sharp peaks at 2850 and 2919 cm^–1^, indicating characteristic symmetric and asymmetric stretching vibration of the ceramide CH_2_ groups (Figure 4a).^48^ Two other peaks, centered at 1632 and 1541 cm^–1^, were observed, which correspond to the amide I and amide II groups. Further, two additional peaks were observed at 3289 and 3412 cm^–1^, indicating amide A and hydroxyl groups of the polar sugar unit of GlcCer (Figure 4a). In contrast, GlcCer assemblies exhibited two less-intense peaks at 2849 and 2918 cm^–1^, corresponding to the ceramide CH_2_ groups and two broad peaks at 1654 cm^–1^ and 1540 cm^–1^, corresponding to the amide I and amide II groups, clearly indicating the presence of intermolecular hydrogen-bonding. Besides, a broad peak was observed at 3680 cm^–1^, indicating that the hydroxyl groups of the polar head and the NH- of amide A of the GlcCer were hydrogen-bonded with the intermolecular hydroxyl group of sphingosines (or glycans) or with the environmental H_2_O (Figure 4b).

Next, PXRD was used to understand the molecular arrangement for formation of lateral domain in the lipid bilayer and lipid–lipid interactions within the GlcCer assemblies.^50,51^ PXRD of the GlcCer monomers exhibited mostly a noisy spectrum (Figure 4c), suggesting that it attained no regular arrangement. However, the PXRD spectrum of GlcCer assemblies displayed five pronounced peaks with the diffraction wave vector, *Q* = 0.22 Å^–1^, 0.34 Å^–1^, 0.45 Å^–1^, 1.42 Å^–1^, and 1.52 Å^–1^, corresponding to the *d*-spacing of 2.75 nm, 1.82 nm, 1.37 nm, 0.44 nm, and 0.41 nm, respectively, indicating a gel phase formation,^52^ possibly having a liquid-crystalline nature, of the GlcCer fibrillar sample (Figure 4d). To gain further molecular level information on the GlcCer fibrils and validate the PXRD result, we performed SAXS and WAXS measurements. We observed that the fibrils exhibited lamellar reflections in the low-angle region (SAXS) with scattering wave vector (*Q*) of 0.11 Å^–1^, corresponding to a lamellar repeat distance (d1), of 5.5 nm (Figure 4e), indicating the repeat distance of a lamellar phase formed by the GlcCer molecules.^52,53^ The different X-ray harmonics (marked with red arrows, for example, *Q* = 0.22 Å^–1^, 0.34 Å^–1^, and 0.45 Å^–1^) observed in SAXS analysis, which were also evident in PXRD, was further indicative of the presence of lamellar stacking. The wide-angle region (WAXS) showed different harmonics (marked with red arrows) and two strong reflections pointing toward real-space spacing of ∼0.44 nm (d2) and ∼0.41 nm (d3) (Figure 4f), characteristic of well-ordered chain packing, indicating the presence of a gel phase.^52−55^ The two pronounced peaks observed in WAXS correspond to the distance between the two polar head groups in lamellar plane (lipid bilayer stacking model in Figure 4g). The fibrillar organization’s dimensions suggest that GlcCer forms a bilayer in which monomers are arranged pairwise in a tail-to-tail configuration with the polar head groups exposed out to the aqueous solvent (Figure 4g).

The average height of the GlcCer fibrils was found to be 29 ± 8 nm (Figure 3), and the lamellar repeat distance (each lipid bilayer) was 5.5 nm (Figure 4e), suggesting that there are approximately 4–6 lipid bilayers stacked together and extended toward the ribbon. The helicity or twist observed in the ribbon structure of the GlcCer fibrils is possibly due to the chiral glucose headgroup, as observed for GalCer aggregates.^46,47^ In addition, we observed weaker correlation peaks in our scattering data (*i*.*e*., peaks not marked with arrows in Figure 4e,f). These represent finer structural details that go beyond the scope of our coarse-grained model (Figure 4g).

### Effect of Preformed GlcCer Assemblies on α-Synuclein Aggregation

**Figure 5:**
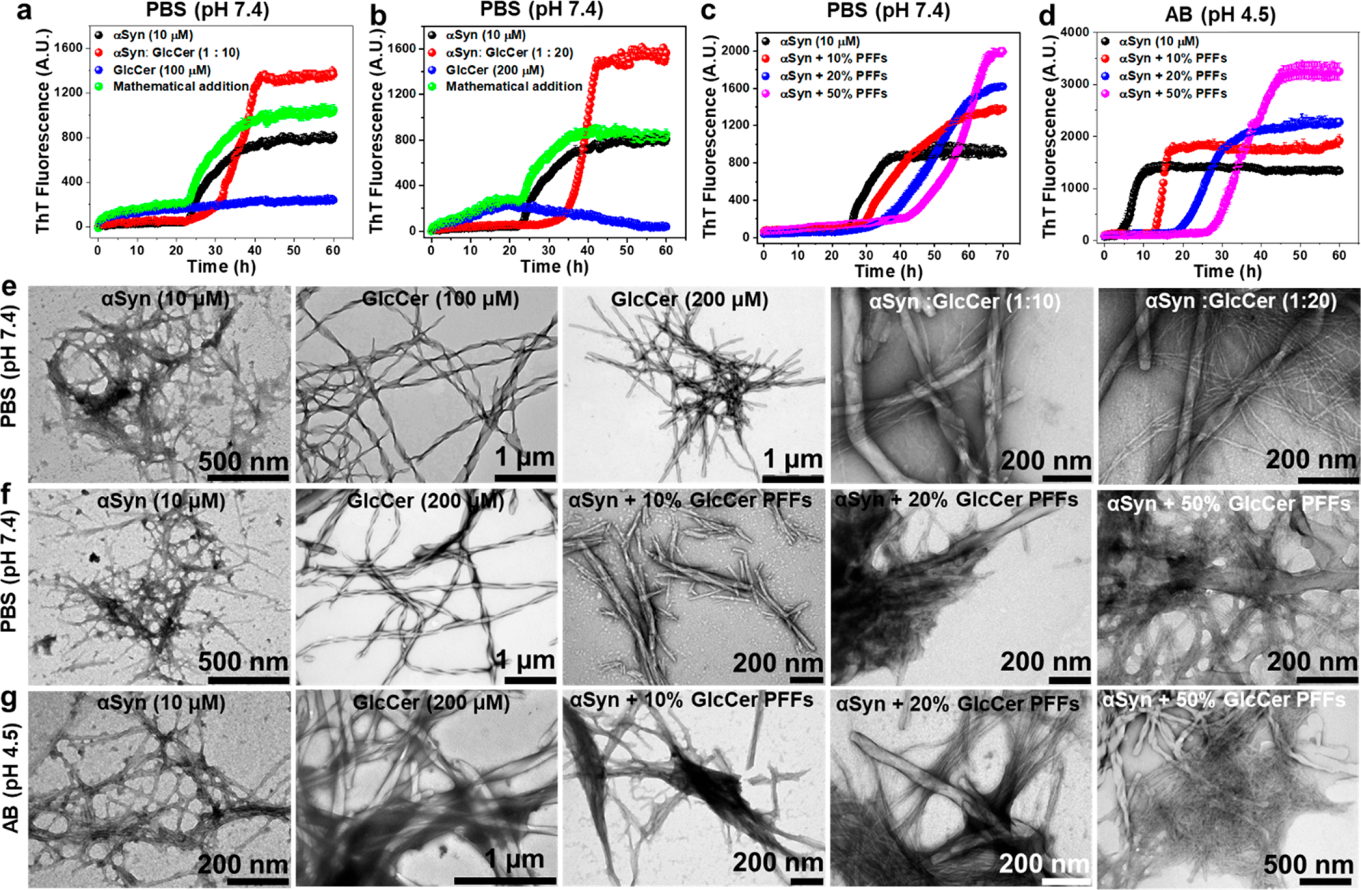
Effect of GlcCer on αSyn aggregation. (a,b) Time-dependent ThT kinetics for the aggregation of αSyn (10 μM), GlcCer (100 μM, 200 μM) and of their coincubated samples or the mathematical addition of the individual kinetic curves. (c,d) Time-dependent ThT kinetics for the aggregation of αSyn (10 μM) in the absence or presence of different doses of GlcCer PFFs at (c) pH 7.4 and (d) pH 4.5. (e) TEM images of aggregated αSyn (10 μM), GlcCer (100 μM or 200 μM), and of their coincubated samples. (f,g) TEM images of αSyn (10 μM) aggregated in the absence or presence of different doses of GlcCer PFFs at (f) pH 7.4 and (g) pH 4.5. All the experiments were repeated at least three times with similar observations.

Amyloid-like assemblies of specific metabolites were found to promote the aggregation of amyloidogenic proteins.^8,10,12^ Interestingly, GlcCer in liposomes was reported to promote aggregation of αSyn.^29,30^ We therefore examined whether free GlcCer assemblies can promote the aggregation of αSyn *in vitro*. In the first series of experiments, as a reference, we coincubated nonaggregated GlcCer (100 μM) with monomeric αSyn (10 μM) in PBS at pH 7.4, and the kinetics of their coaggregation was monitored for 60 h by ThT assay, after which the samples were analyzed by TEM. Untreated monomeric αSyn and GlcCer only served as controls. ThT fluorescence of untreated αSyn increased gradually with a lag phase of ∼22 h and reached a plateau in 50 h (Figure 5a). The control GlcCer (100 μM) was also found to aggregate with time at faster kinetics; however, the extent of ThT fluorescence was much lower than that of αSyn (Figure 5a). The αSyn sample coincubated with GlcCer, aggregated at a slower rate (lag phase of ∼25 h) than the untreated αSyn. However, the extent of aggregation (end point level of ThT fluorescence) was much greater than that of untreated αSyn (Figure 5a). To verify that the increase of ThT signal upon coincubation was due to the effect of GlcCer on αSyn aggregation rather than a sum of their independent aggregation signals, we summed the ThT kinetic curves of untreated αSyn and GlcCer only. We observed that the generated sum curve has a different kinetic pattern than the experimental curve observed from their coincubation (Figure 5a), and the latter has a much higher end point ThT signal. This result indicates that GlcCer has a distinct effect on αSyn aggregation kinetics and the formation of mature αSyn fibrils. Similar results were observed when the concentration of GlcCer was increased from 100 μM to 200 μM, yet a longer lag-phase (∼34 h) with a greater extent of ThT signal than the untreated αSyn was observed (Figure 5b). TEM micrographs revealed that the control samples of untreated αSyn or GlcCer-only formed densely fibrillar assemblies, where the fibrils of the former were much smaller and thinner (∼7–10 nm) than the latter (∼50–60 nm) (Figure 5e). Notably, upon coincubation, the two types of fibrils colocalized. Taken together, the ThT and TEM results indicate promotion of free GlcCer on αSyn aggregation in agreement with the report that GlcCer in liposomes enhances the aggregation αSyn.^29,30^

Next, we examined how GlcCer aggregates affect αSyn aggregation. To that end, GlcCer (200 μM) was first aggregated for 10 h to form fibrils as described above (Figure 1a,d). Then monomeric αSyn (10 μM) was coincubated at various ratios with the GlcCer preformed fibrils (PFFs) for different durations, and their coaggregation was monitored using ThT binding (Figure 5c,d) and TEM (Figure 5f,g). The ThT kinetic assay revealed that at pH 7.4, control untreated αSyn aggregated slowly with a lag phase of ∼23 h and reached a plateau within 50 h (Figure 5c). In the presence of 10% v/v of GlcCer PFFs, the rate of aggregation of αSyn was reduced, with a slightly longer lag phase of 27 h; however, the aggregation reached a plateau with a much higher ThT signal than in the absence of the GlcCer PFFs (Figure 5c). Increasing the dose of GlcCer PFFs (20–50% v/v) resulted in a longer lag-phase (32–42 h), yet higher level of ThT fluorescence than in the presence of 10% (v/v) GlcCer PFFs. The morphology of the samples at the end point of the ThT assay, examined by TEM, revealed that in either dose of GlcCer PFFs, the grids were more densely populated with fibrils of αSyn than in their absence (Figure 5f). These results suggest that GlcCer fibrils induce the aggregation of αSyn.

GlcCer accumulates in the lysosome whose milieu is acidic. We next examined the effect of the GlcCer PFFs on αSyn aggregation *in vitro* at a low pH of 4.5, near the lysosomal condition. At pH 4.5 (acetate buffer), αSyn (10 μM) aggregated much faster, with a shorter lag phase of ∼5 h, than at physiological pH (lag phase ∼25 h), in accord with previous studies.^56,57^ In the presence of different doses of GlcCer PFFs (10–50% v/v), the rate of αSyn aggregation was reduced, with an increased lag phase of 13–27 h (Figure 5d); however, the extent of the ThT signal at plateau was 2–3-fold higher than that of αSyn aggregated alone. To quantify amyloid formation by αSyn in the presence or absence of GlcCer PFFs, we performed two additional independent ThT experiments (sets 2 and 3) along with set 1 (Figure 5d) at identical conditions, and the % amyloids were calculated from the end-point ThT fluorescence. We have averaged all the data of each of the three sets to compare the end-point ThT fluorescence of αSyn aggregation in the presence of GlcCer PFFs *vs* in their absence. We observed that the end-point ThT fluorescence of αSyn aggregation in the presence of 10%, 20%, or 50% (v/v) PFFs of GlcCer PFFs was significantly higher (set 1: 143 ± 7%, 170 ± 4%, 242 ± 7%; set 2: 132 ± 9%, 157 ± 9%, 224 ± 13%; set 3: 137 ± 13%, 163 ± 22%, 230 ± 27%; respectively) than in their absence (100%), considering the end-point ThT fluorescence of untreated αSyn as 100% (Figure S13). These results suggest that the GlcCer PFFs induce αSyn fibril formation. The formation of fibrils at the end point of the ThT assay, analyzed by TEM (Figure 5g), revealed a clear fibrillar network when αSyn aggregated alone. In the presence of various doses of GlcCer PFFs, the grids clearly contained both GlcCer fibrils (thicker morphology of ∼50 nm in diameter) and αSyn fibrils (thinner morphology of ∼10 nm in diameter), and the latter were more densely populated than in grids containing αSyn aggregated alone (Figure S14).

To examine the cytotoxicity of the αSyn aggregates in the absence or presence of 50% (v/v) GlcCer PFFs, we incubated αSyn with GlcCer PFFs at pH 4.5 and 37 °C for 60 h. At different time points, samples were collected and applied to SH-SY5Y cells for 12 h, after which cell viability was compared to that of cells incubated with untreated αSyn fibrils (Figure S15). As controls, we also treated cells with 50% (v/v) PFFs of GlcCer only and with untreated αSyn fibrils. The viability of control untreated cells was set as 100%. In this XTT assay, we observed that the untreated αSyn fibrils reduced viability to 70 ± 3%, whereas the PFFs of GlcCer only exhibited 77 ± 5% of cell viability. The αSyn aggregates formed upon incubation in the presence of 50% (v/v) PFFs, for 2 h, 5 h, 15 h, 25 h, 30 h, and 60 h caused cell viabilities of 68 ± 2%, 37 ± 3%, 34 ± 5%, 43 ± 4%, 54 ± 3%, and 59 ± 3%, respectively. The viability of cells incubated with αSyn aggregates that formed in the presence of 50% (v/v) GlcCer PFFs was much lower than that of cells incubated with αSyn aggregates that formed in the absence of GlcCer PFFs, indicating that the former were more toxic than the latter. Interestingly, αSyn incubated with GlcCer PFFs for 2–25 h showed a much higher toxicity (evident in lower cell viability) in comparison to the samples incubated for 30–60 h as well as untreated αSyn fibrils. Since the concentration of PFFs was same in all of the treated samples, the contribution of cytotoxicity by these GlcCer PFFs themselves would be same. Therefore, the higher cytotoxicity observed for the 2–25 h incubated samples is likely due to the increased relative concentration of αSyn oligomers *vs* mature fibrils, which was decreased upon further incubation (30–60 h) concomitantly with the increase in the relative concentration of mature αSyn fibrils. Indeed, the samples incubated with PFFs for 30–60 h contained mainly mature αSyn fibrils (as evident from ThT and TEM analysis, Figure 5d,g) and exhibited lower cytotoxicity than the samples incubated for 2–25 h and higher than untreated αSyn fibrils. These results indicate that αSyn samples (either fibrils or oligomers) incubated with 50% PFFs of GlcCer were more cytotoxic than the mature untreated α-synuclein fibrils. The higher cytotoxicity of the αSyn aggregates incubated with GlcCer PFFs for 2–25 h provides further support for the suggestion that the GlcCer PFFs stabilize toxic αSyn oligomers.

**Figure 6:**
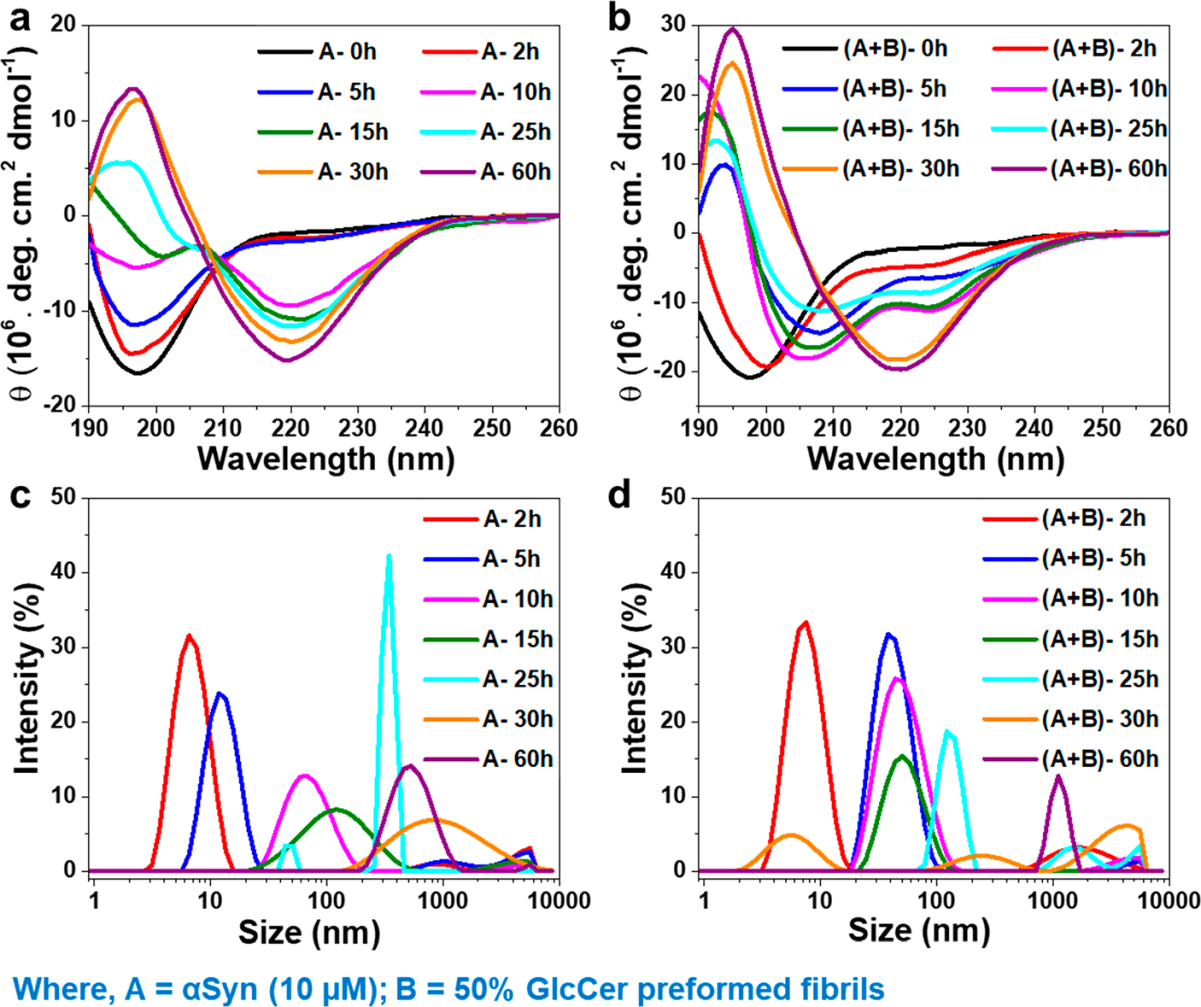
Effect of preformed GlcCer fibrils on the stabilization of αSyn oligomers. CD spectra recorded at different time points for the aggregation of A (αSyn (10 μM)) in the (a) absence or (b) presence of B (50% (v/v) GlcCer PFFs). Size distribution curve measured by DLS analysis at different time points of A (αSyn (10 μM)) aggregation in the (c) absence or (d) presence of B (50% (v/v) GlcCer PFFs).

To gain insight into the nature of the species formed upon incubation of αSyn (10 μM) with GlcCer PFFs (50% v/v) at near lysosomal pH (4.5), we employed circular dichroism (CD) spectroscopy and dynamic light scattering (DLS) at various time points during the 0–25 h of coincubation. At time 0, the control sample of αSyn alone exhibited a negative CD peak at 200 nm, suggesting random coil conformation (Figure 6a).^58^ Upon 5 h of coincubation, αSyn displayed two negative CD peaks at ∼200 nm and ∼ 220 nm and a positive peak at ∼195 nm, reflecting a mixture of β-sheet and random coil conformations, as previously reported.^58^ Upon further incubation up to 15 h or longer, the control sample presented complete β-sheet conformation as only one negative maximum was observed at ∼220 nm and a positive peak at ∼195 nm, suggesting fibril formation by αSyn. In the presence of GlcCer PFFs (50% v/v), at time 0, αSyn exhibited a negative peak at 200 nm, suggesting random coil conformation and possibly the presence of monomeric protein conformation (Figure 6b). Upon 5–25 h of coincubation, two negative peaks were observed at ∼208 nm and ∼222 nm, suggesting α-helical conformation with a mixture of some β-sheet structure, conceivably due to the presence of soluble oligomeric structure of αSyn according to previous reports.^59,60^ Until 25 h of coincubation, the α-helical conformation was the primary species observed; however at later time-points, CD spectra indicated conversion into β-sheet rich conformation, reflecting delayed amyloid fibril formation compared to αSyn alone. To avoid the chiral interference, CD spectra of treated αSyn were subtracted from the CD spectra of 50% PFFs of GlcCer alone (Figure S16a).

For DLS analysis, samples were collected from the CD experiment at different time points, centrifuged at 50,000*g*, and the soluble portion of the reaction mixtures was examined. The sample of untreated αSyn at 2 h of incubation exhibited a hydrodynamic diameter of ∼8 nm. With incubation time, the average diameter was increased to ∼15 nm, ∼55 nm, ∼110 nm, ∼320 nm, ∼720 nm, and ∼540 nm at 5 h, 10 h, 15 h, 25 h, 30 h, and 60 h, respectively (Figure S7c). In contrast, the hydrodynamic diameter of the sample of αSyn coincubated with 50% (v/v) GlcCer PFFs was smaller ∼7 nm, ∼35 nm, 40 nm, 45 nm, 122 nm, 255 nm, and 905 nm at 2 h, 5 h, 10 h, 15 h, 25 h, 30 h, and 60 h, respectively (Figure 6d). These results suggest that the GlcCer fibrils possibly stabilized the αSyn aggregates at an oligomeric conformation and slowed down their progress toward the formation of larger assemblies. However, upon coincubation for longer periods (30–60 h), the αSyn oligomers probably further self-assembled to form large fibrillar assemblies as evident in DLS by the reduced intensity of smaller size species and enhanced intensity of the larger size species, corroborated by ThT assay and TEM images (Figure 5d,g). As a control, the size distribution function and respective autocorrelation functions (ACFs) from DLS analysis of GlcCer PFFs was also examined (Figure S16b,c).

The size distribution results obtained from DLS were supported by the ACFs of the scattered light, extracted from DLS analysis (Figure S17). In the absence of GlcCer PFFs, the αSyn aggregates quickly as evident from the rapid change in initial slope of ACF, which reflects the presence of small size oligomers,^61^ from 2 h to 5 h and the slope of the ACF remains stable after 10 h (Figure S17a). Likewise, the ACF curves at 10–60 h exhibited higher values in the time domain at the midpoint of the transition, indicating larger aggregates compared to the values of 2 h and 5 h samples. These results indicate the short life (5 h) of soluble oligomers and rapid formation of large aggregates (fibrils) by the untreated αSyn. In contrast, αSyn samples treated with GlcCer PFFs were found to aggregate slowly, as the initial slope of the ACF curve changes gradually from 2 h to 25 h and thereafter remains stable (Figure S17b). In addition, the ACF curves from 30 h to 60 h exhibited higher values in the time domain at the midpoint of the transition than the curves at 2–25 h, suggesting slower formation of larger aggregates (fibrils) by αSyn in the presence of GlcCer PFFs. These results suggest that GlcCer PFFs stabilize the oligomers of αSyn. Collectively, all of these *in vitro* results suggest that the preformed GlcCer fibrils promoted aggregation of αSyn.

### Mitigation of GlcCer Aggregation by Inhibitors of Proteinaceous Amyloids

Accumulation of GlcCer in the lysosome is a hallmark of GD, and we observed that accumulated GlcCer forms amyloid-like fibrils *in vitro*. Therefore, inhibition of self-assembly of GlcCer may have a therapeutic value. To this end, several small molecules (EGCG, NQTrp, RA, allantoin), which were reported as effective inhibitors of aggregation of various amyloidogenic proteins,^62,63^ were examined for their effect on self-assembly of GlcCer at pH 4.5 (near lysosomal pH).

**Figure 7:**
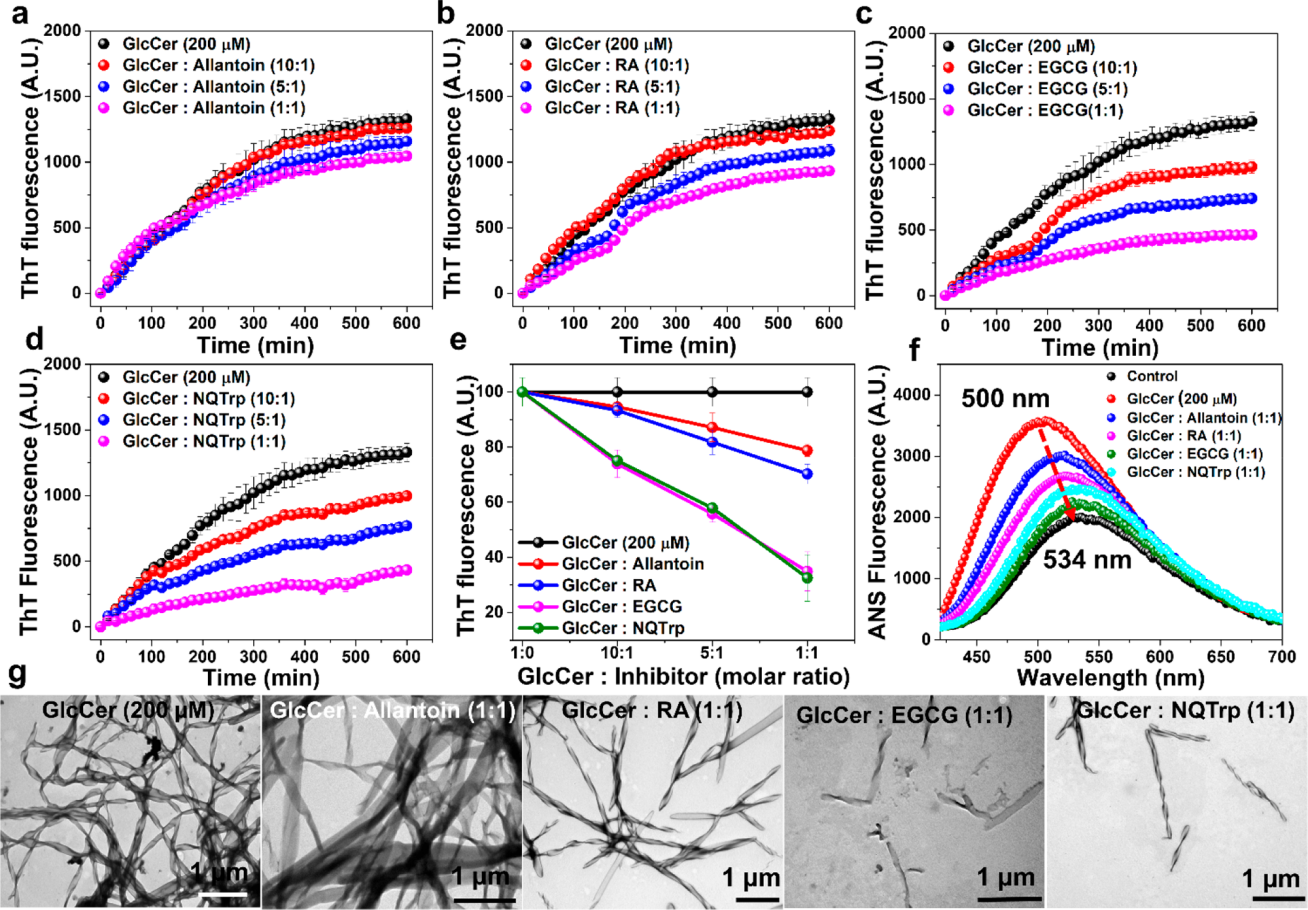
Inhibition of GlcCer aggregation by amyloid inhibitors. (a–d) Time-dependent ThT kinetics for the aggregation of GlcCer in the absence or presence of different molar ratios (GlcCer: inhibitor = 10:1, 5:1, and 1:1 molar ratio) of (a) allantoin, (b) RA, (c) EGCG, and (d) NQTrp. (e) Dose-dependent ThT assay for the aggregation of GlcCer in the absence or presence of different molar ratios of the inhibitors. (f) ANS binding assay of aggregated GlcCer samples in the absence or presence of a 1-fold molar ratio of the candidate inhibitors. (g) TEM images of GlcCer aggregates in the absence or presence of a 1-fold molar ratio of the candidate inhibitors. Experiments were repeated at least three times with similar observations.

The kinetics of GlcCer aggregation in the absence or presence of different molar ratios of the inhibitors (GlcCer: inhibitor 10:1, 5:1, and 1:1) were monitored by ThT binding assay (Figure 7a–e). In the absence of inhibitors, ThT fluorescence of GlcCer (200 μM) aggregation was rapidly increased with time as observed before (Figures 1a and 2d), suggesting the formation of amyloid structures. ThT signal was reduced with time in a dose-dependent manner when EGCG or NQTrp were coincubated with GlcCer, indicating substantial aggregation inhibition (Figure 7c,d). However, a minimal decline in ThT fluorescence was noted in the presence of RA and allantoin, indicating that these molecules were less effective in inhibiting aggregation of GlcCer even at 5-fold molar excess of the inhibitors (Figure 7a,b). To complement these observations, ANS binding assay was employed at the end of the ThT kinetics analysis for the GlcCer samples treated with a 1-fold molar excess of the different inhibitors. Untreated GlcCer exhibited a maximum of ANS emission signal at ∼500 nm, whereas the control ANS only showed an emission maximum at ∼534 nm (Figure 7f). The overt blue shift from 534 to 500 nm and with a higher intensity indicates binding between the hydrophobic patches of GlcCer with the ANS dye (Figure 1c). However, GlcCer samples treated with a 1-fold molar excess of the different inhibitors showed redshift from 500 to 530 nm, indicative of the lower binding ability of the GlcCer fibrils with ANS in comparison with the untreated GlcCer samples (Figure 7f), suggesting mitigation of aggregation by the tested molecules with EGCG and NQTrp being more effective.

To corroborate the ThT and ANS results, TEM analysis was performed on the samples at the end of the kinetics experiments. Control GlcCer alone could form a densely fibrillar network (Figures 7g and S18). The density of the fibrils declined in a dose-dependent manner in the presence of EGCG or NQTrp, but not when incubated with RA or allantoin, in agreement with the ThT and ANS results. These observations indicate that certain *bona fide* inhibitors of aggregation of amyloidogenic proteins can mitigate the formation of amyloid-like aggregates of GlcCer.

We further examined the effect of these tested inhibitors to disaggregate the PFFs of GlcCer in pH 4.5 at 37 °C. GlcCer alone was first allowed to aggregate for 10 h to form fibrils, then different doses of the inhibitors (GlcCer:inhibitors = 1:1 and 1:5) were added to the PFFs and incubated for additional 10 h. Thereafter, the ThT fluorescence signal was recorded and the % amyloid was calculated, considering the ThT fluorescence of untreated GlcCer PFFs as 100% (Figure S19). We observed that neither of the tested doses (1-fold or 5-fold molar excess) can reduce the ThT signal, suggesting the inefficiency of the tested inhibitors to disaggregate the PFFs of GlcCer even at higher dose (5-fold molar excess). TEM analysis (Figure S20) was performed to support the ThT results. Collectively, these results indicate that the candidate inhibitor molecules were unable to disaggregate the PFFs of GlcCer.

To verify whether the ability to form amyloid-like twisted fibrils is exclusive to GlcCer or any lipid can exhibit such property, we separately incubated GlcCer and ceramide (Cer) in AB pH 4.5 at 37 °C for 10 h and monitored their aggregation propensity using ThT kinetics, ANS binding, and TEM study. We observed that the ThT intensity increased rapidly with time in GlcCer solutions in all the tested concentrations (10–200 μM) (Figure S21a). In contrast, the change in ThT fluorescence with time was much lower in the solution of Cer, which is almost comparable to the background ThT fluorescence (Figure S21a). The end-point ThT fluorescence intensity was calculated, and we noticed that the intensity increased up to 10 times in the GlcCer solution in comparison to the control ThT (background fluorescence) (Figure S21b). Whereas the ThT fluorescence intensity of Cer showed almost equal or 1.5 times increased fluorescence than the background fluorescence (Figure S21b). These results indicate higher binding affinity and aggregate formation by GlcCer than Cer.

ANS binding assay was further performed, and it was observed that the samples containing GlcCer exhibited a blue shift with higher intensity in comparison to the ANS alone (Figure S21c). In contrast, Cer did not show any blue shift, yet exhibited a slight increase in intensity. These results indicate that GlcCer strongly binds with ANS dye than Cer. We also examined the morphology of these lipid assemblies using TEM, and we observed a clear fibrillar species in GlcCer sample, but no such fibrillar structure was noticed in Cer samples (Figure S22). Collectively, these results indicate that Cer did not aggregate and form fibrils, but GlcCer aggregates and forms amyloid-like fibrils.

## Conclusions

Our results indicate that molecules of free GlcCer self-assemble spontaneously *in vitro*, very much like classical proteinaceous amyloids. The resulting fibrils bind amyloid-specific dyes, including ThT, ANS, and Congo red, suggesting that they are rich in hydrophobic structures, and their morphology exhibits typical amyloid-like attributes. TEM, AFM, and SEM analyses revealed that the fibrils are organized as twisted helical ribbons, ∼52 nm wide, ∼29 nm high, and twist (pitch) of ∼184 nm. These structural values resemble the previously reported twisted helical GlcCer fibrils obtained from GD patients’ brains (25–50 nm wide, ∼5 μm long).^45^ These structural parameters of GlcCer are also very similar to those reported for *in vitro* generated GalCer fibrils, where 20:1^Δ11^GalCer aggregates formed helical ribbons that have a right-handed twist with an average width of 30–35 nm and helical pitches of 80–90 nm.^46,47,64^

Our combined FTIR and X-ray scattering results indicate that the free GlcCer spontaneously forms a lamellar phase in which the long-chain nonpolar tails of GlcCer monomers interact with each other to organize pairwise in a tail-to-tail configuration, and the polar glycan head groups remain exposed to the aqueous medium. The AFM analysis showed that the average width of the GlcCer fibrils was 29 ± 8 nm. In SAXS analysis, the lamellar repeat distance was observed as 5.5 nm, indicating that the GlcCer forms a typical lipid bilayer arrangement with 4–6 lipid bilayers stacked together and extended toward the ribbon. It appears that the chirality in GlcCer does not allow its monomers to stabilize and pack parallel to their nearest neighbors, but rather at a nonzero twist angle to them, leading the entire lamellar phase to twist into a helical ribbon.^65,66^ Such ribbon-like structures not only have been shown for synthetic lipids but have also been described for various biological lipids that form bilayer tubules, notably cerebrosides such as GlcCer and GalCer.^45−47^

Mazulli *et al*. have observed that *in vitro* incubation of a mixture of two different lipids, purified GlcCer and brain phosphatidylcholines (PCs), at PC:GlcCer 9:1 to 1:3 molar ratios, under acidic conditions influenced the aggregation of αSyn.^30^ However, as the proportion of GlcCer was increased (from 25 to 75%), while keeping the total lipid amount constant (PC 25%, GlcCer 75%), the kinetic profile of αSyn fibril formation was altered by delaying the formation of insoluble ThT-positive αSyn fibrils, extending the lag time from 2 to 16 h. Along these lines, our results indicate that at physiological conditions (pH 7.4), GlcCer assemblies induced αSyn fibril formation with a comparable delay of 4–19 h. However, at acidic conditions (pH 4.5), the delay in the aggregation process was more profound, 13–27h. SEC and SDS-PAGE analyses by Mazulli *et al*. indicate that during the lag phase (between 1 and 5 h), the induced αSyn soluble oligomeric species appeared to become more abundant. In contrast, in the control samples (αSyn only), the proportion of oligomers and monomers was reduced, consistent with their recruitment into insoluble fibrils. They suggest that GlcCer has stabilized the formation of a soluble assembly competent species during the observed lag phase. We observed a similar effect by the GlcCer amyloid-like aggregates. Using immune-EM with syn505 antibody that preferentially detects misfolded αSyn, they detected αSyn species colocalized with GlcCer tubular structures, suggesting a direct interaction between GlcCer and αSyn that facilitates its fibril formation.

Similar results were subsequently reported *in vivo*. GSL accumulation in human induced pluripotent stem neurons promoted αSyn aggregation into toxic conformations.^29^ Immunofluorescence and immuno-EM showed that GlcCer and αSyn colocalized in these cells, suggesting that a direct interaction may facilitate αSyn conformational conversion inside neurons. Toxic aggregates of αSyn could be produced in these cells in the absence of *GBA1* mutations, yet with reduced GCase1 activity and accumulation of GSL, suggesting that GSLs alone can induce αSyn pathology. GSLs could induce structural conversions of αSyn also in a mouse model.^29^

The free GlcCer aggregates used in the present study, similar to the previously reported lipid mixture, possibly stabilized the oligomerization of αSyn and reduced the process of secondary nucleation, which might delay the onset of its aggregation. Subsequently, the soluble oligomeric species started assembling for maturation, leading to the formation of higher-ordered aggregates and exhibited a stronger ThT signal than the signal observed when αSyn was alone. These findings can provide a mechanistic explanation for the apparent prevalence of PD among GD patients.

Certain small molecules, including EGCG, RA, NQTrp, and allantoin, effectively inhibited the aggregation of proteinaceous amyloids (including Aβ, αSyn, and tau) and ameliorated pathology and symptoms of the related diseases in animal models.^62,63^ The observation that, among the tested molecules, EGCG and NQTrp inhibited amyloid-like aggregation of GlcCer should be helpful for comparative studies in GD and GD-PD *in vivo*. We speculate that the hydroxyl groups of the glycan head of GlcCer may form hydrogen bonds with the tested inhibitors more strongly than its self-assembly process, thus leading to the inhibition of GlcCer aggregation. In addition, the aromatic ring of the tested molecules, for example, NQTrp or EGCG, may strongly interact with the cluster of carbon atoms of the hexose ring through the energetically favorable CH−π stacking.^67,68^

In conclusion, the present study describes the characterization of amyloid-like aggregates of GlcCer, which exhibit a twisted ribbon-like structure. The GlcCer aggregates were found to delay the aggregation of αSyn *in vitro* and increased the stabilization of the oligomers known to be a major cause of PD pathogenesis. Finally, we showed that certain inhibitors of protein aggregation could inhibit also the aggregation of GlcCer. Our findings provide a mechanistic insight of GlcCer aggregation and its linkage with αSyn pathology, suggesting a target for therapy and drug development toward GD and PD.

## Experimental Methods

### Materials

All of the chemicals used were of the highest purity grade. Black and transparent, flat-bottomed polystyrene nonbinding 96-well plates were purchased from Corning, USA. Glucosylceramide (GlcCer, C18:1 glucosyl(β) ceramide (d18:1/18:1(9Z)); *M*_W_ 726.08; catalog no. 860548P) and ceramide (Cer, (d18:1/18:1(9Z)); *M*_W_ 563.94; catalog no. 860519P) were purchased from Avanti Polar lipids, USA. α-Synuclein was purchased from rPeptide, USA (catalog no. S-1001-2). Molecular biology grade ultrapure water and all the solvents and reagents used for the XTT assay were purchased from Biological Industries (Israel) and Biological Industries, Israel. All other chemicals were purchased from Sigma-Aldrich (Rehovot, Israel).

### Stock Preparation

GlcCer powder was dissolved in 100% DMSO to prepare 100 mM stock which was further diluted to desired concentrations with different buffer solutions (phosphate buffer saline (PBS) of pH 6.5 or 7.4, acetate buffer (AB) of pH 4.5, and double disstilled or deionized water (DDW)) as required. Commercially available αSyn was monomerized by a 10 min pretreatment with HFIP, and the solvent was evaporated using a Speed Vac. The resulted thin film was dissolved in PBS/AB and sonicated for 5 min. The strength of the working buffer system for all assays was 100 mM. Concentration of the protein was determined using Nano drop (calculated according to ε_280_ of 5960 M^–1^ cm^–1^) and adjusted to 50 μM concentration as a stock solution. Stock solutions of Thioflavin T (ThT, 4 mM) were prepared in DDW. Stock solutions of the tested inhibitor molecules (10 mM) were prepared separately in DMSO and diluted with PBS/AB before use. The stock solutions of the GlcCer and protein were used immediately after preparation, and for each assay, a fresh stock was prepared separately.

### Thioflavin T Fluorescence Assay

ThT fluorescence assay was carried out in a similar manner as described previously to monitor the protein aggregation.^69^ In brief, to monitor the aggregation kinetics of GlcCer, the stock solutions were diluted in 100 μL wells in a 96-well black plate so that the final mixture contained 10–200 μM of GlcCer and 20 μM ThT in 100 mM PBS (or in AB). For the aggregation kinetic of αSyn, the final concentration was used as 10 μM from the stock of 50 μM in 100 mM PBS (or in AB). For the αSyn aggregation kinetics, continuous agitation was used. For the inhibition of GlcCer aggregation, the tested inhibitor molecules (GlcCer:inhibitor = 10:1, 5:1, 1:1) were added separately to the designated wells, and kinetics of aggregation was monitored by ThT fluorescence at 37 °C. To monitor the effect of GlcCer PFFs on αSyn aggregation, GlcCer was first allowed to aggregate alone for 10 h to obtain PFFs, and then different doses of the preaggregated GlcCer fibrils were added to the designated well and coincubated with αSyn for 60–70 h. The data were collected (in triplicates) using Infinite M200 microplate reader (Tecan, Switzerland), with measurements taken at 15 min intervals. Excitation and emission wavelengths of ThT were 440 and 485 nm, respectively. The plate was incubated at 37 °C. Measurements were performed as independent triplicates. Three recorded values were averaged, and background measurements for buffer containing only ThT were subtracted.

### Turbidity Assay

GlcCer was dissolved in 100% DMSO to prepare a stock concentration of 100 mM. The stock GlcCer was diluted to a desired concentration with PBS (or AB) and incubated at 37 °C. For the turbidity measurement, we examined the absorbance spectra at 350 nm, using an Infinite M200 microplate reader (Tecan, Switzerland) and polypropylene 96-well plates (Corning) in 100 μL volume. Plates were shaken gently for 5 s prior to each measurement cycle and incubated at 37 °C. All optical densities were measured at 350 nm wavelength, and their first measurement (OD (*T*_0_)) was subtracted in order to eliminate background absorbance of the samples.

### ANS Fluorescence Assay

A 20 μL aliquot of GlcCer aggregates taken from turbidity assay was mixed with 100 μM 8-anilinonaphthalene-1-sulfonic acid (ANS) in the desired buffer (PBS or acetate buffer). ANS fluorescence intensities were measured with excitation at 380 nm and emission between 400 and 700 nm using Infinite M200 microplate fluorescence reader (Tecan, Switzerland).

### Transmission Electron Microscopy

Samples (10 μL) were placed for 2 min over 400 mesh copper grids covered with carbon-stabilized Formvar film (Electron Microscopy Sciences, Hatfield, PA). Excess fluid was removed, and the grids were negatively stained with 2% uranyl acetate solution (10 μL) for 2 min. Then, the excess fluid was removed and allowed to dry for 5 min. The samples were viewed using a JEM-1400 TEM (JEOL), operated at 80 kV.

### Scanning Electron Microscopy

Samples (10 μL) were placed on glass slides and left to air-dry under ambient conditions for 12 h. Samples were then coated with Au for conductance and viewed using a benchtop scanning electron microscope (JEOL, Tokyo, Japan) operating at 20 kV.

### Atomic Force Microscopy

AFM imaging was performed by depositing 10 μL solutions onto freshly cleaved V1 grade mica (Ted Pella, Redding, CA, USA). The samples were allowed to dry under ambient conditions for 12 h. The samples were imaged using AFM (JPK Instruments AG) performed with Nano Wizard 3 with 5 N/m spring constant tips and a resonance frequency of ∼150 kHz in soft tapping mode. AFM analysis was performed on different areas for each sample, and the GlcCer fibrils parameters (twist, diameter and height) were measured using the Gwyddoin 2.56 software.

### Congo Red Birefringence

Congo red powder was dissolved in 80% aqueous ethanol to prepare a saturated stock solution. The solution of aggregated GlcCer (5 μL) was mixed with 5 μL of saturated Congo red solution and incubated for 10 min. The suspension (10 μL) was then drop casted over a glass side, and the samples were dried in air and kept in a desiccator before birefringence analysis. The samples were viewed at 20× magnification with a Nikon Eclipse TI polarizing microscope. Digitized images were obtained using a Nikon DS Ri1 digital camera.

### ThT Staining and Fluorescence Imaging

Aggregate GlcCer samples (10 μL), obtained from turbidity assay, were mixed with 10 μL of ThT dye (20 μM), and the mixture was drop casted over glass slide followed by drying under ambient conditions for 12 h. Then the dried samples were viewed under fluorescence microscope (Nikon Eclipse TI polarizing microscope) using a GFP filter.

### Cell Cytotoxicity Experiments

The SH-SY5Y human neuroblastoma cell line (2 × 10^5^ cells/mL) was cultured in 96-well tissue microplates (100 μL/well) and allowed to adhere overnight at 37 °C. The stock of GlcCer (in DMSO) was dissolved in DMEM:Nutrient mixture F12 (Ham’s) (1:1) (Biological Industries, Israel) at different concentrations (1–200 μM) for 6 h to generate GlcCer assemblies (based on ThT results). 1% DMSO was used as the vehicle. The negative control was prepared as a medium without GlcCer or DMSO and treated in the same manner. 100 μL of medium with or without GlcCer aggregates was added to each well. Following incubation for 24 h at 37 °C, cell viability was evaluated using the 2,3-bis(2-methoxy-4-nitro-5-sulfophenyl)-2H-tetrazolium-5-carboxanilide (XTT) cell proliferation assay kit (Biological Industries, Israel) according to the manufacturer’s instructions. Briefly, 100 μL of the activation reagent was added to 5 mL of the XTT reagent, followed by the addition of 50 μL of activated-XTT solution to each well. After 2 h of incubation at 37 °C, color intensity was measured using an ELISA microplate reader at 450 and 630 nm. Results are presented as mean and the standard error of the mean. Each experiment was repeated at least three times.

### Fourier-Transform Infrared Spectroscopy

FTIR spectroscopy was performed with 30 μL of GlcCer samples (monomers or aggregates) of 200 μM concentration. Monomers were obtained by dissolving GlcCer powder in chloroform/methanol (2:1) solvent. Aggregates of GlcCer were prepared in the same manner as described above for the turbidity assay. The samples were deposited onto disposable KBr IR sample cards (Sigma-Aldrich), which were then allowed to dry under vacuum. Transmission infrared spectra were collected using a Nexus 470 FTIR spectrometer (Nicolet) with a deuterated triglycine sulfate detector. Measurements were performed using the atmospheric suppression mode by averaging 64 scans in 2 cm^–1^ resolution.

### Powder X-ray Diffraction

Powder GlcCer samples (monomers and aggregates) were poured inside a glass capillary of 0.5 mm in diameter. Monomers were obtained by dissolving GlcCer powder in chloroform/methanol (2:1) solvent and evaporated under argon to get solid powder. The aggregated samples were prepared by incubating 200 μM of GlcCer in DDW for 12 h, and the assembled fibers were lyophilized to obtain fluffy powder. XRD was collected in symmetric Bragg–Brentano geometry with Cu K_α_ radiation source (wavelength of 1.54 Å) on a BrukerD8Discover X-ray diffractometer equipped with a one-dimensional LynxEye detector based on compound silicon strip technology.

### Small and Wide-Angle X-ray Scattering

The aggregated GlcCer samples at 30 mg/mL concentration were measured in 1.5 mm diameter sealed quartz capillaries. WAXS/SAXS measurements were performed using an in-house solution X-ray scattering system, with a GeniX (Xenocs) low divergence Cu K_α_ radiation source (wavelength of 1.54 Å) and a scatterless slits setup.^70^ Two-dimensional scattering data with a wave vector amplitude (*Q*) range of 0.06–2 Å^–1^ at a sample-to-detector distance of about 230 mm were collected on a Pilatus 300 K detector (Dectris), and radially integrated using MATLAB (MathWorks) based procedures (SAXSi). Background scattering data were collected from buffer solution alone. The background-subtracted scattering correlation peaks were fitted using a Gaussian with a linearly sloped baseline. For each sample, time-resolved correlation peaks position, intensity, and width were extracted.

### *In Vitro* Coincubation of Recombinant α-Synuclein with GlcCer Assemblies

Recombinant αSyn (rPeptide, USA) was dissolved in PBS (pH 7.4) or acerate buffer (pH 4.5) to a concentration of 50 μM. The monomeric protein was mixed with the either nonaggregated GlcCer (100 μM and 200 μM) or preformed GlcCer assemblies (10% v/v, 20% v/v, or 50% v/v), taken from 500 μM stock solution, to a final concentration of 10 μM αSyn. The samples were then incubated at 37 °C, and the kinetics of αSyn aggregation in the absence or presence of different GlcCer samples was monitored by ThT and TEM following a similar process as described above.

### Circular Dichroism Spectroscopy

To analyze the secondary structure of the αSyn aggreagtes in the absence or presence of different doses of preaggregated GlcCer, 300 μL of the samples were taken in a cuvette (path length 1 mm), and CD spectra were then recorded on a Chirascan spectrometer between the range of 190 and 260 nm, and the background was subtracted from the CD spectra. Since DMSO absorbs at far UV range, stocks of GlcCer was prepared in methanol for this assay.

### Dynamic Light Scattering

DLS^71,72^ measurements were performed with the different αSyn samples (in the absence or presence or GlcCer assemblies) on a Malvern nano zetasizer (Malvern, UK) using a laser source with λ = 633 nm and a detector at a scattering angle of θ = 173°. Prior to DLS measurement, different αSyn samples, with or without GlcCer assemblies, were ultracentrifuged for 30 min (Beckman Optima TLXl Benchtop Ultracentrifuge, TLA-120.1 Rotor Package, Fixed Angle, Titanium, 14 × 0.5 mL) at 50,000*g* to remove the larger αSyn aggregates and GlcCer assemblies. The supernatant solution was further ultracentrifuged two more times to completely remove the larger aggregates, and the final supernatant samples containing αSyn soluble aggregates were used for DLS measurement. Prior to the analysis, samples were filtered through a 0.2 μm PVDF membrane. The samples were placed in a disposable cuvette and held at the corresponding temperature during the analysis. For each sample, the analyses were recorded three times with 11 subruns using the multimodal mode. The *Z*-average diameter was calculated from the correlation function using Malvern technology software. Representative autocorrelation functions (ACFs) were directly adapted from the DLS data obtained for each sample.

## Supporting Information Available

Chemical structure of GlcCer; TEM and AFM images of GlcCer fibrils; TEM images of GlcCer aggregates to monitor the critical concentration of fibril formation; visual appearance of the GlcCer aggregates of different concentrations at pH 7.4; TEM images of GlcCer fibrils at different concentrations, different time points, at different temperature and in 80% aqueous EtOH; end-point ThT fluorescence and morphology under TEM of GlcCer aggregates at different concentrations and pHs; calculation of structural parameters analyzed from AFM images of GlcCer fibrils; end-point ThT fluorescence of αSyn aggregation in the presence of different doses of GlcCer PFFs; TEM images of αSyn aggregates in the presence of 50% (v/v) GlcCer PFFs; cell viability assay (XTT) of αSyn aggregates in the presence of GlcCer PFFs; CD and DLS analysis of control 50% (v/v) GlcCer PFFs; ACF, obtained from DLS measurement, of αSyn oligomers in the presence or absence of GlcCer fibrils; TEM images of the inhibited GlcCer fibrils in the presence of different dose of the tested inhibitors; bar diagram showing end-point fluorescence and TEM images of GlcCer PFFs in the presence of different doses of the tested inhibitors; ThT kinetics, ANS binding assay, and TEM images showing the aggregation propensity of GlcCer, but not Cer (PDF)
