# Supplementary material for: Glucosylceramide Associated with Gaucher Disease Forms Amyloid-like Twisted Ribbon Fibrils That Induce α-Synuclein Aggregation
Source: ACS Nano. 2021 Jul 2;15(7):11854–68. doi: 10.1021/acsnano.1c02957 (PMC8397424; doi:10.1021/acsnano.1c02957)
Supplement: Supplementary file 1 — nn1c02957_si_001.pdf [file nn1c02957_si_001.pdf]

Supporting Information

Glucosylceramide Associated with Gaucher Disease Forms Amyloid-like Twisted Ribbon Fibrils That Induce α-Synuclein Aggregation

Ashim Paul,[1] *Guy Jacoby,*[2] *Dana Laor Bar-Yosef,*[1] *Roy Beck,*[2] *Ehud Gazit,*[1,3] *Daniel Segal*[1,4*]

[1]Department of Molecular Microbiology and Biotechnology, Shmunis School of Biomedicine and Cancer Research, Tel Aviv University, Ramat Aviv, Tel Aviv 6997801, Israel.

[2]The Raymond and Beverly Sackler School of Physics and Astronomy, The Center for Nanoscience and Nanotechnology, and the Center for Physics and Chemistry of Living Systems, Tel Aviv University, Tel Aviv 69978, Israel

[3]Department of Materials Science and Engineering, Iby and Aladar Fleischman Faculty of Engineering, Tel Aviv University, Tel Aviv, 69978, Israel

[4]Sagol Interdisciplinary School of Neuroscience, Tel Aviv University, Ramat Aviv, Tel Aviv 6997801, Israel.

[*]Corresponding Author

Daniel Segal

Department of Molecular Microbiology and Biotechnology, Shmunis School of Biomedicine and Cancer Research, Tel Aviv University, Ramat Aviv, Tel Aviv 6997801, Israel

E-mail: dsegal@post.tau.ac.il; phone: ++972-3-640-9835; fax: ++972-3-640-9407

Supporting Figures

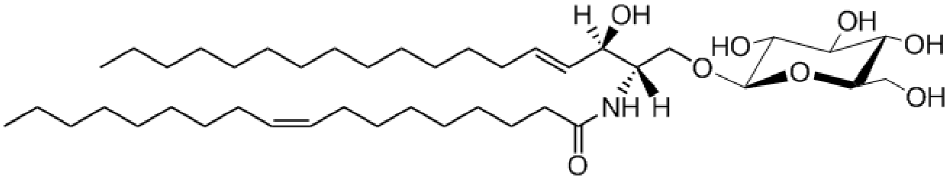

Figure S1. Chemical structure of the Glucosylceramide (GlcCer, D-glucosyl-ß-1,1'-N-oleoyl-D-erythro-sphingosine or C18:1 Glucosyl(ß) Ceramide (d18:1/18:1(9Z))) used in the present study. Molecular Formula $C_{42}H_{79}NO_8$ (MW 726.079).

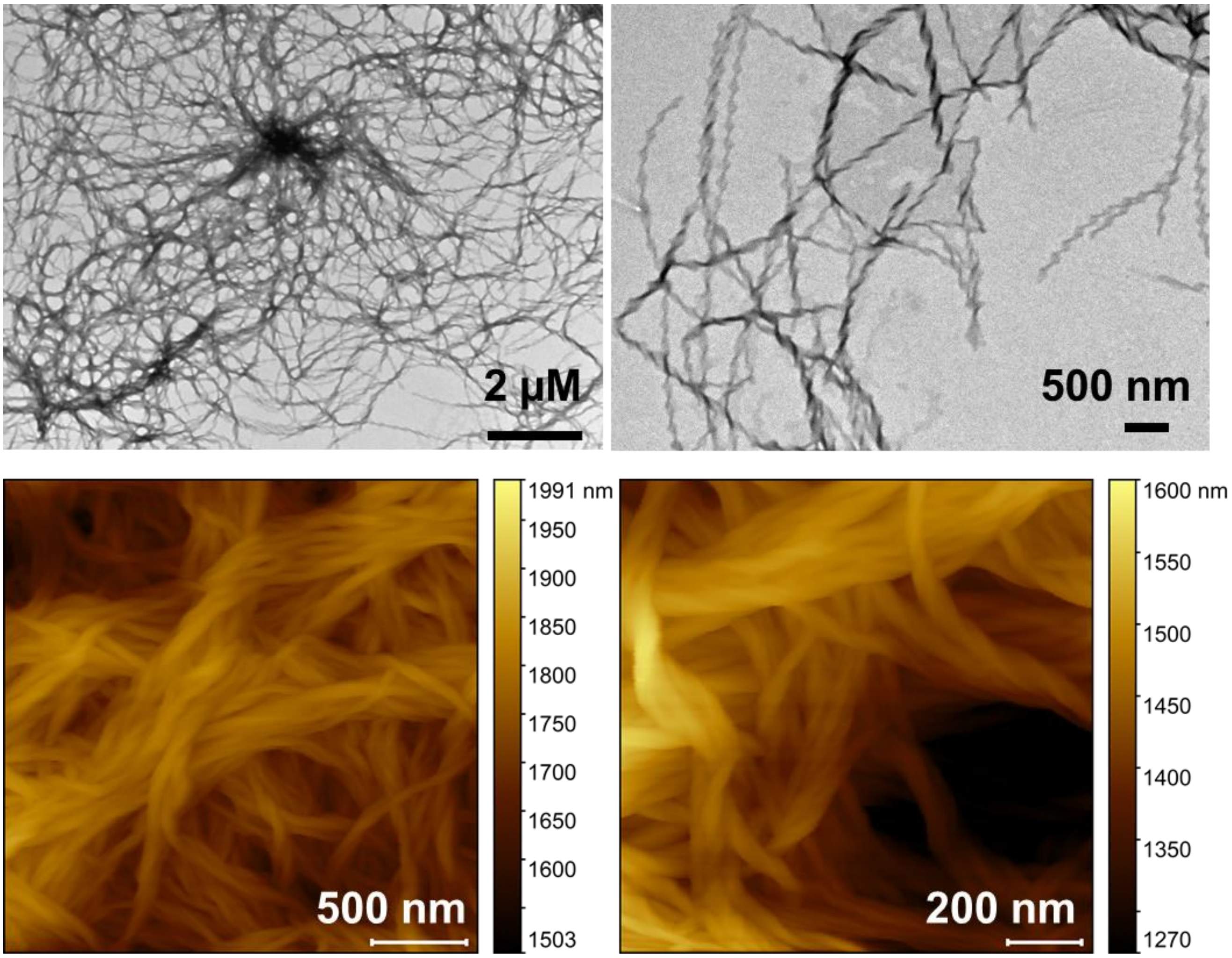

Figure S2. TEM (top panel) and AFM (bottom panel) images of GlcCer aggregates. Images were taken after 10 h of incubation of GlcCer (100 µM) in PBS pH 7.4 at 37 °C.

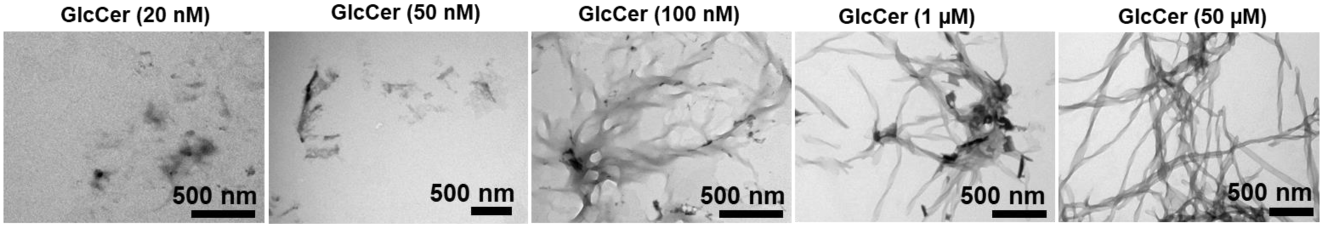

Figure S3. TEM images of GlcCer incubated at different concentrations (20 nM to 50 μM) in PBS pH 7.4. All the samples were incubated for 10 h at 37 ºC prior to the sample preparation for TEM imaging.

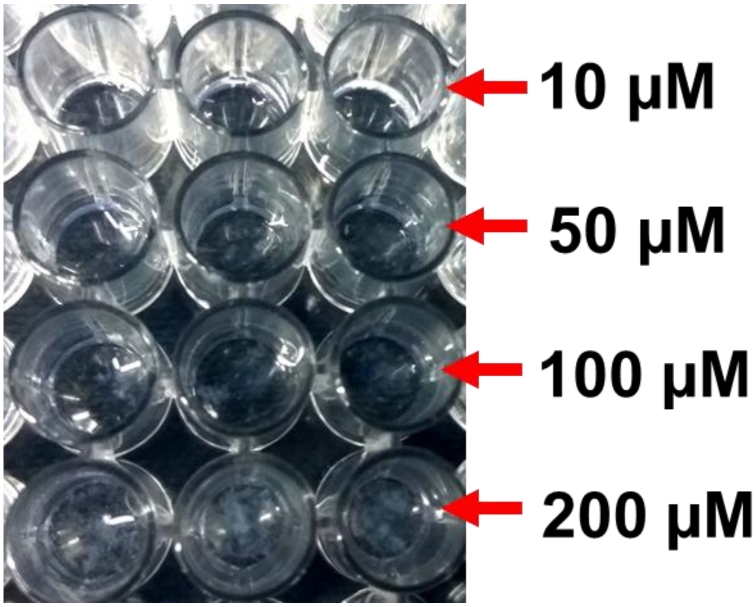

Figure S4. Samples showing turbidity of GlcCer at different concentrations. Image was taken after 10 h of incubation of GlcCer at different concentrations in PBS pH 7.4 at 37 °C.

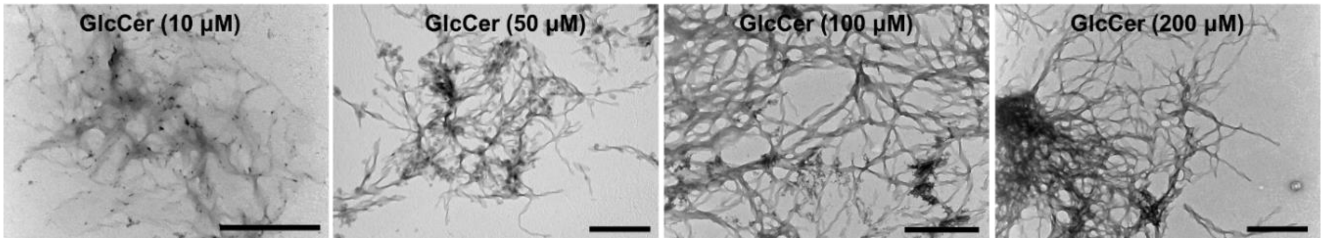

Figure S5. TEM images of GlcCer aggregates at different concentrations (10 - 50 μM). Images were taken after at the end-point of ThT fluorescence.

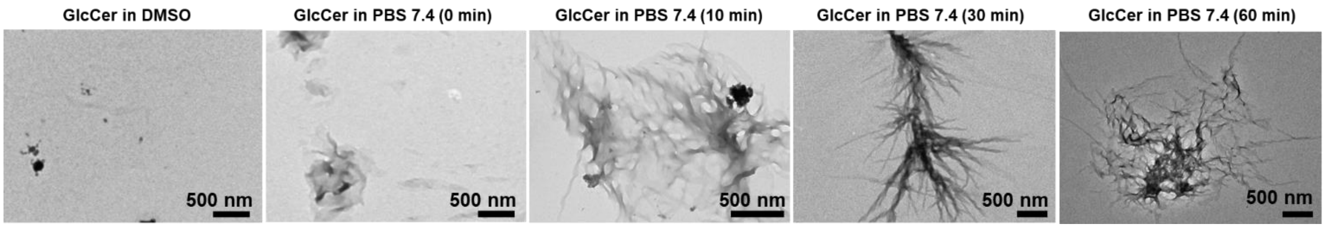

Figure S6. TEM images of GlcCer (100 µM) in DMSO and in PBS pH 7.4. Images were taken at different time points after dilution in PBS from the original stock of GlcCer (10 mM) in DMSO.

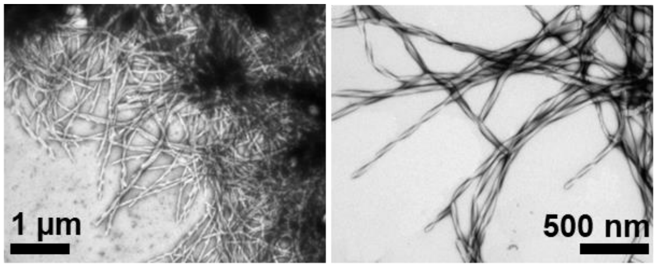

Figure S7. TEM images of GlcCer (100 µM) in PBS pH 7.4. Images were taken after 60 days incubation of GlcCer at 37ºC.

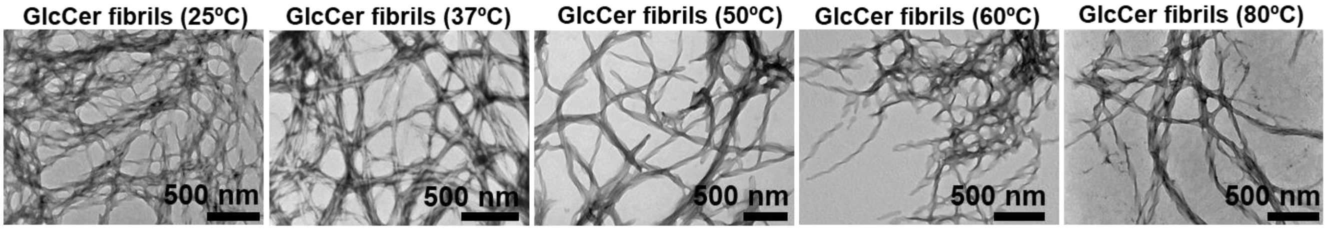

Figure S8. TEM images of GlcCer (100 µM) fibrils incubated at different temperatures. GlcCer (100 µM) monomers were first incubated for 10 h to generate fibrils and these preformed fibrils were further incubated for 10 h at different temperatures before the images were taken.

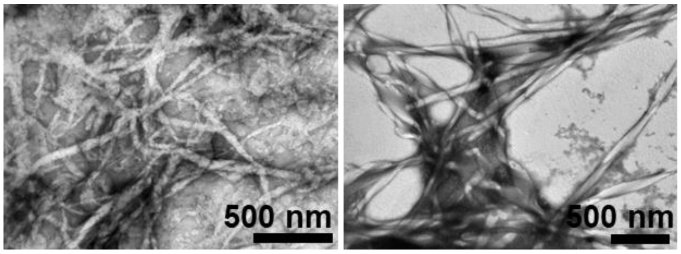

Figure S9. TEM images of GlcCer (100 µM) fibrils in 80% aqueous EtOH. GlcCer (200 µM) was first incubated for 10 h in PBS pH 7.4 and then diluted with equal volume of 80% aqueous EtOH to obtain a final concentration of 100 µM. The final mixture was incubated for an additional 1h and images were taken thereafter.

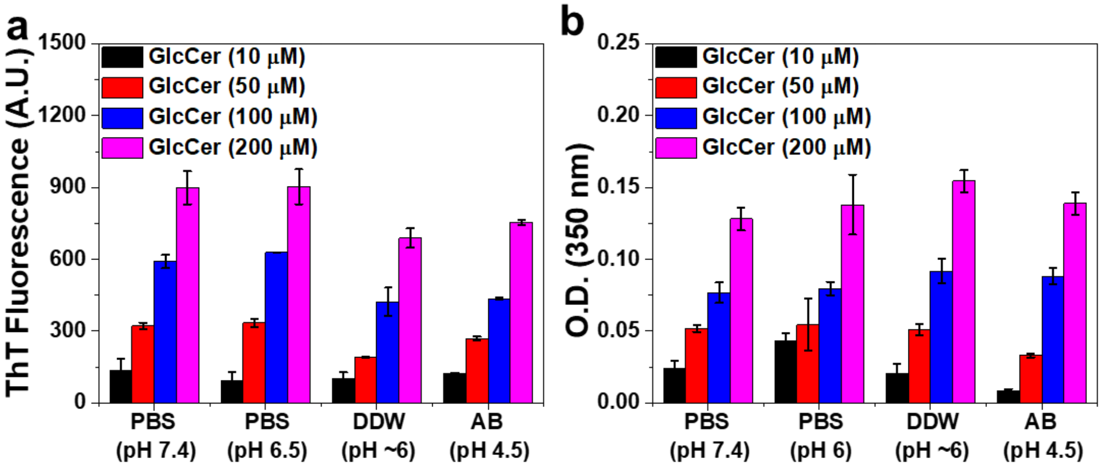

Figure S10. Bar diagram showing the end-point ThT fluorescence (a) and absorbance at 350 nm (b) for the aggregation of different concentrations of GlcCer at various pHs. Experiments were repeated at least three times with similar observations.

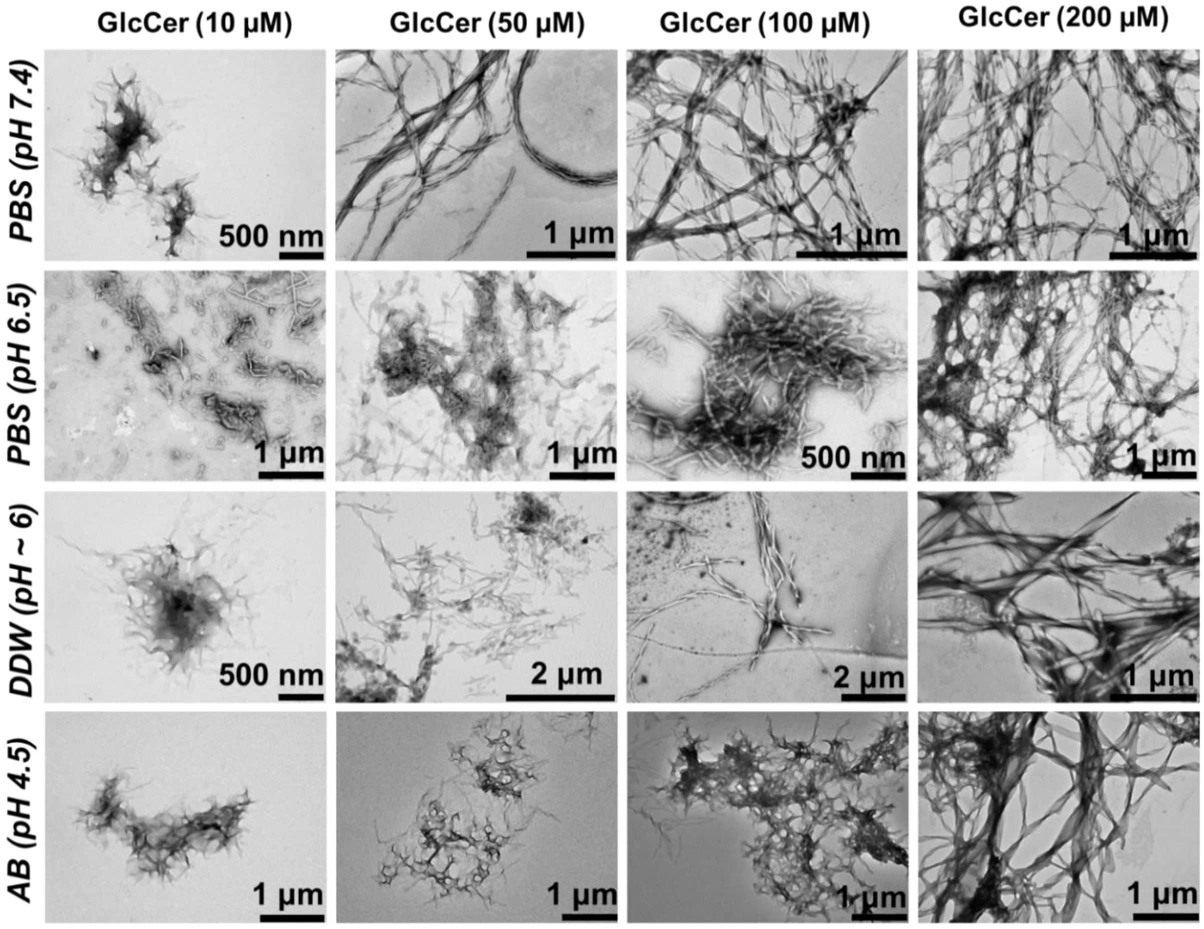

Figure S11. TEM images showing the fibril formation of different concentrations of GlcCer at various pHs. Experiments were repeated at least three times with similar observations.

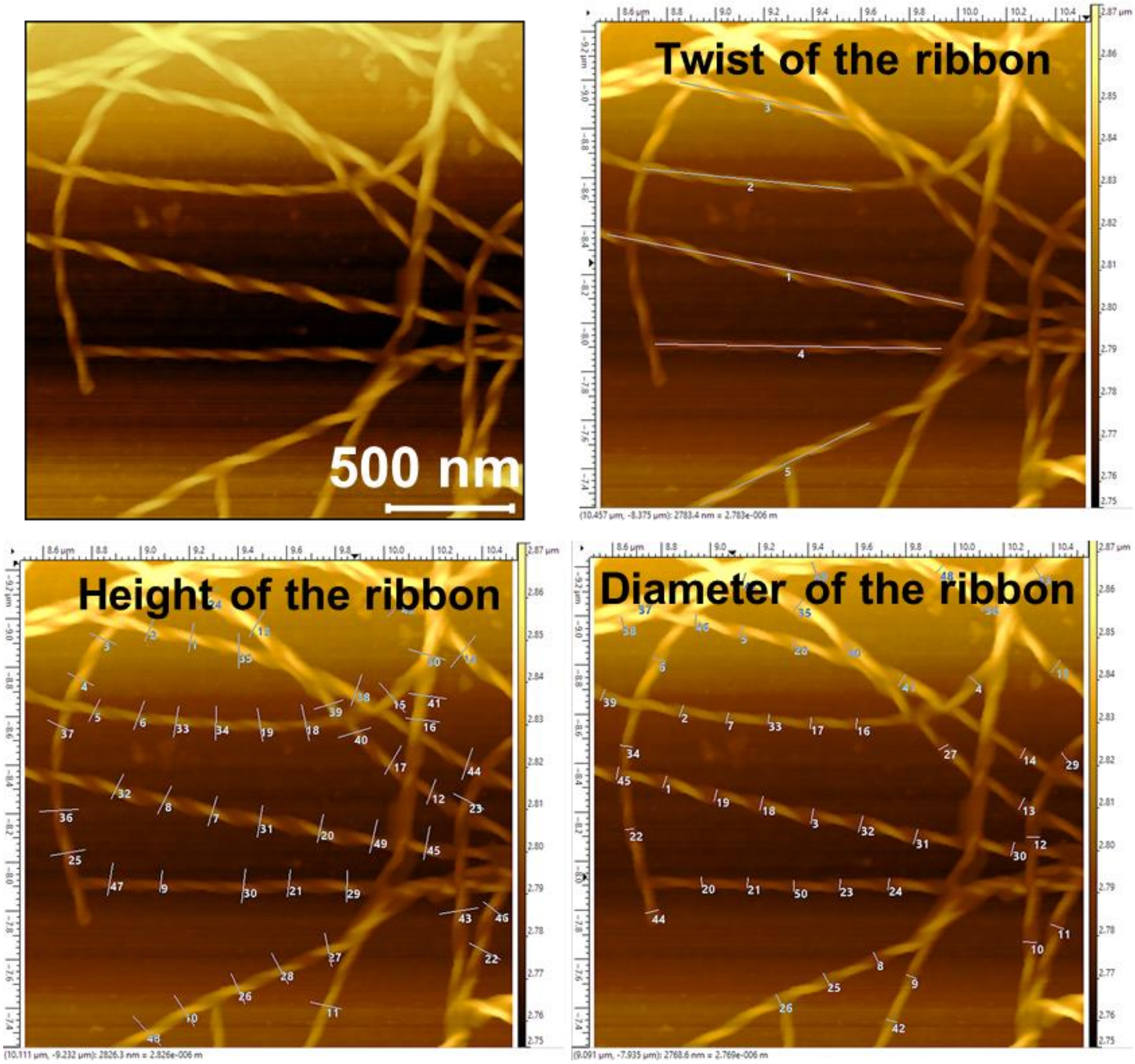

Figure S12. AFM images showing the fibril formation by GlcCer (100 µM) in DDW. Different cross sections were selected to calculate the average twist, width and height of each fibrils using Gwyddoin software.

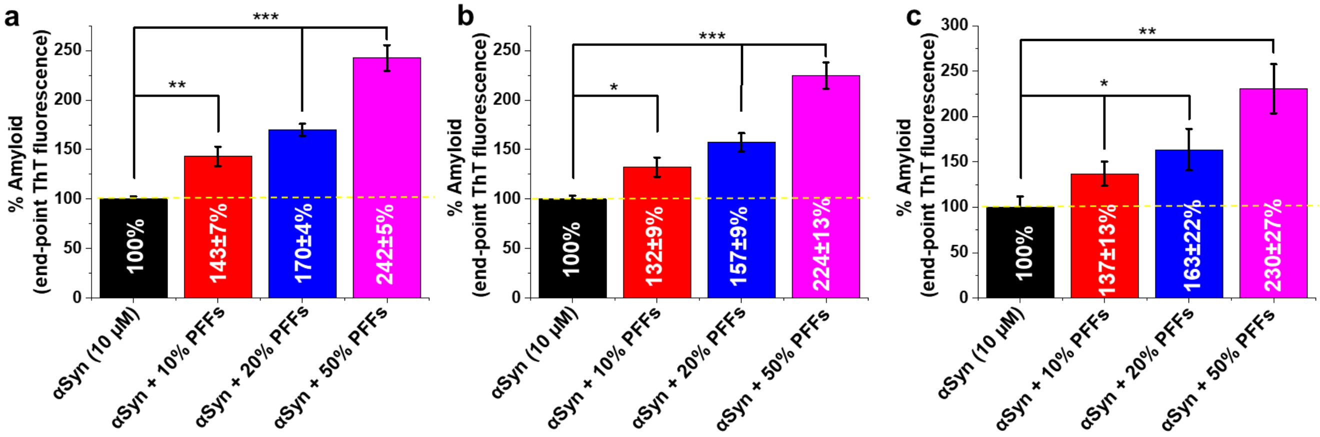

Figure S13. The end-point ThT fluorescence of αSyn aggregation in the absence or presence of 10%, 20% or 50% (v/v) PFFs of GlcCer, set 1 (a), set 2 (b) and set 3 (c). P-values: *p<0.05, **p<0.005 and ***p<0.001.

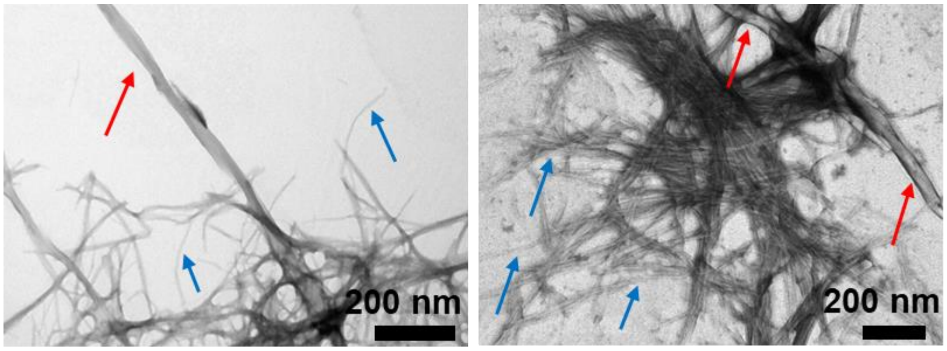

Figure S14. TEM images of αSyn (10 µM) in the presence of 50% (v/v) PFFs of GlcCer. Red arrows indicated GlcCer fibrils (thicker, ~50 nm diameter) and blue arrows indicated αSyn fibrils (thinner, ~10 nm diameter). Samples were incubated in AB pH 4.5 for 60 h prior to the image collection.

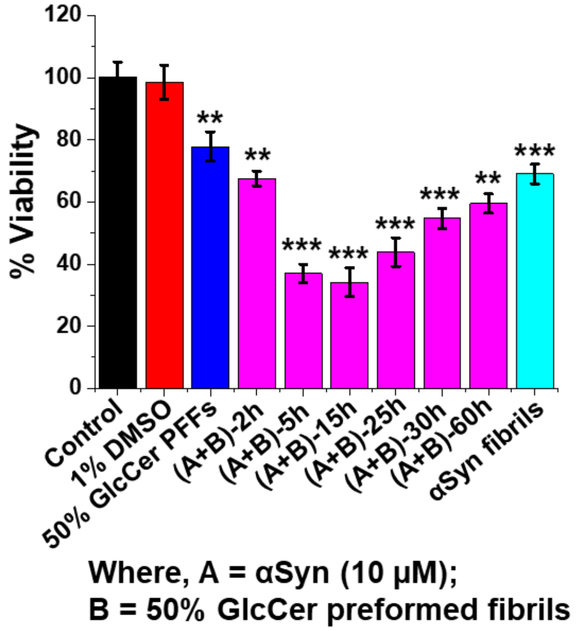

Figure S15. The effect of αSyn aggregates formed in the presence of 50% (v/v) GlcCer PFFs for different durations, on cell viability of SH-SY5Y cell line evaluated. Confluent cells were incubated with the various samples for 12 h and the % cell viability was measured using XTT assay. Untreated cells were used as control and set to 100% viability. Results are average of 6 replicates (n = 6, ±SD) and are expressed as a percentage of control cells. Significance (**p<0.005) and (***p<0.001).

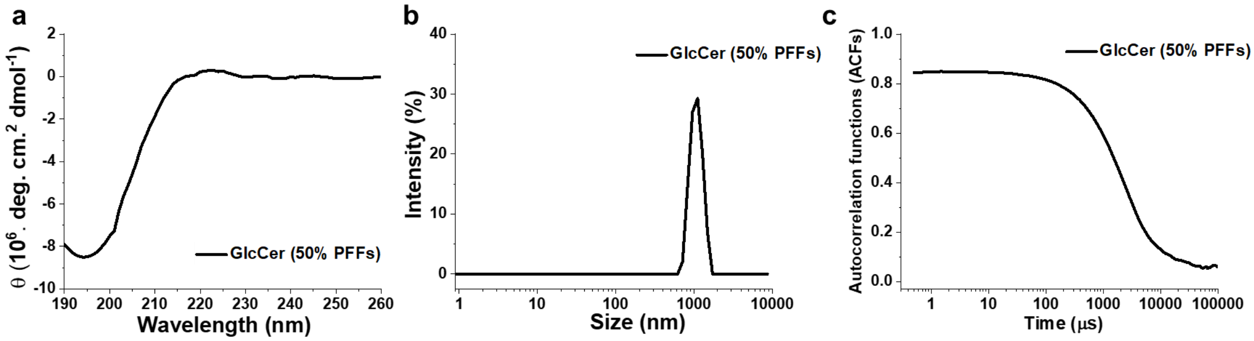

Figure S16. (a) CD spectra; (b) size distribution curve and (c) respective autocorrelation function obtained from DLS analysis of 50% (v/v) GlcCer preformed fibrils.

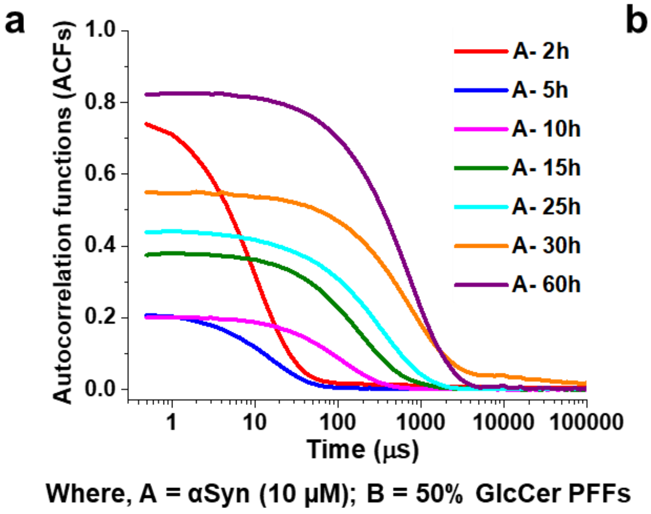

Figure S17. DLS measurement of A (αSyn (10 µM)) aggregation in the absence (a) or presence (b) of B (50% (v/v) GlcCer PFFs). Representative autocorrelation functions (ACFs) were directly adapted from the DLS results obtained for each sample.

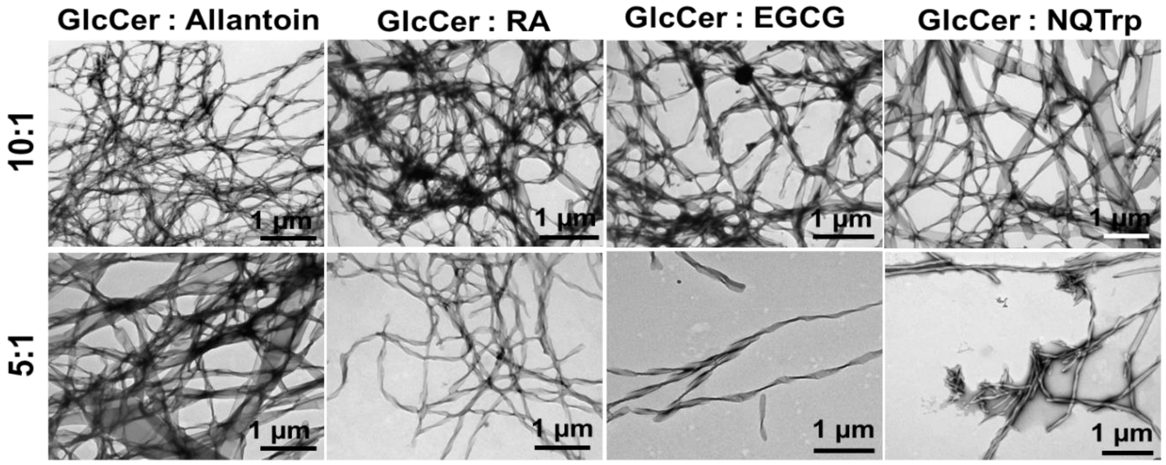

Figure S18. TEM images of GlcCer aggregates in the presence of different doses (GlcCer:inhibitor = 10:1 and 5:1) of Allantoin, RA, EGCG and NQTrp. Experiments were repeated at least three times with similar observations.

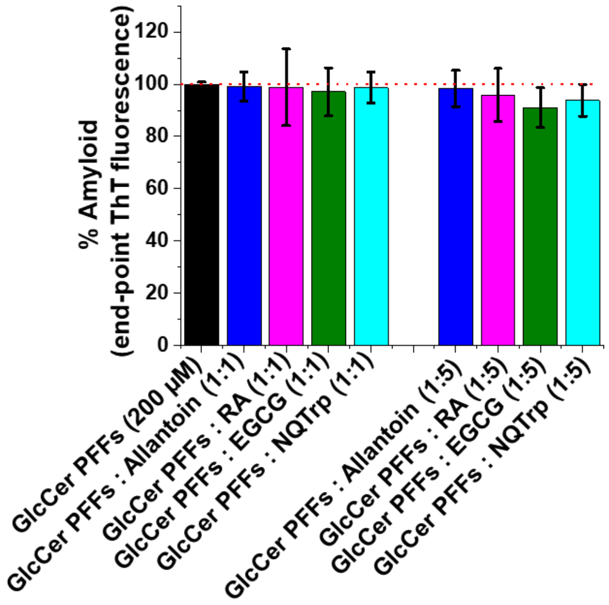

Figure S19. Disaggregation of preformed GlcCer fibrils by amyloid inhibitors. ThT fluorescence for the disaggregation of GlcCer PFFs in the absence or presence of different molar ratios (GlcCer : inhibitor = 1:1 and 1:5 molar ratio) of Allantoin (blue), RA (magenta), EGCG (green) and NQTrp (cyan). GlcCer alone was first allowed to aggregate in AB pH 4.5 at 37 ºC for 10 h to form fibrils, then different doses of the inhibitors were added and incubated for additional 10 h. ThT fluorescence was recorded after 20 h (10+10). Experiments were repeated at least three times with similar observations.

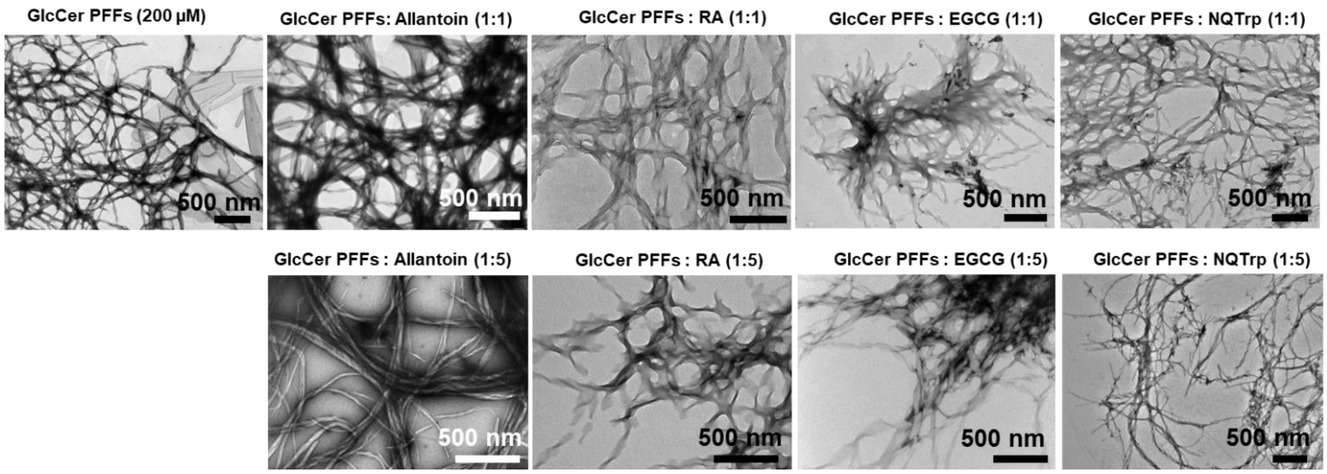

Figure S20. TEM images of GlcCer PFFs incubated in the absence or presence of 1-fold and 5-fold molar excess of the candidate inhibitors.

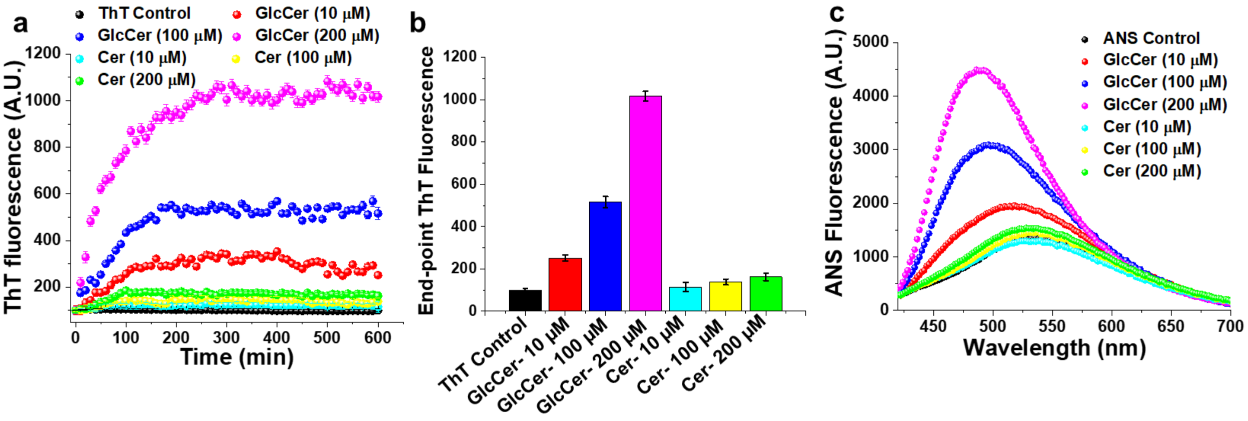

Figure S21. GlcCer but not Cer aggregate and form amyloid-like fibrils. (a) Time-dependent ThT kinetics and (b) end-point ThT fluorescence for the aggregation of GlcCer and Ceramide (Cer) at different concentrations (10-200 μM). Samples were incubated in AB pH 4.5 at 37ºC for 10 h. (c) ANS binding assay of the 10 h incubated GlcCer and Cer samples incubated at different concentrations for 10 h. Experiments were repeated at least three times with similar observations.

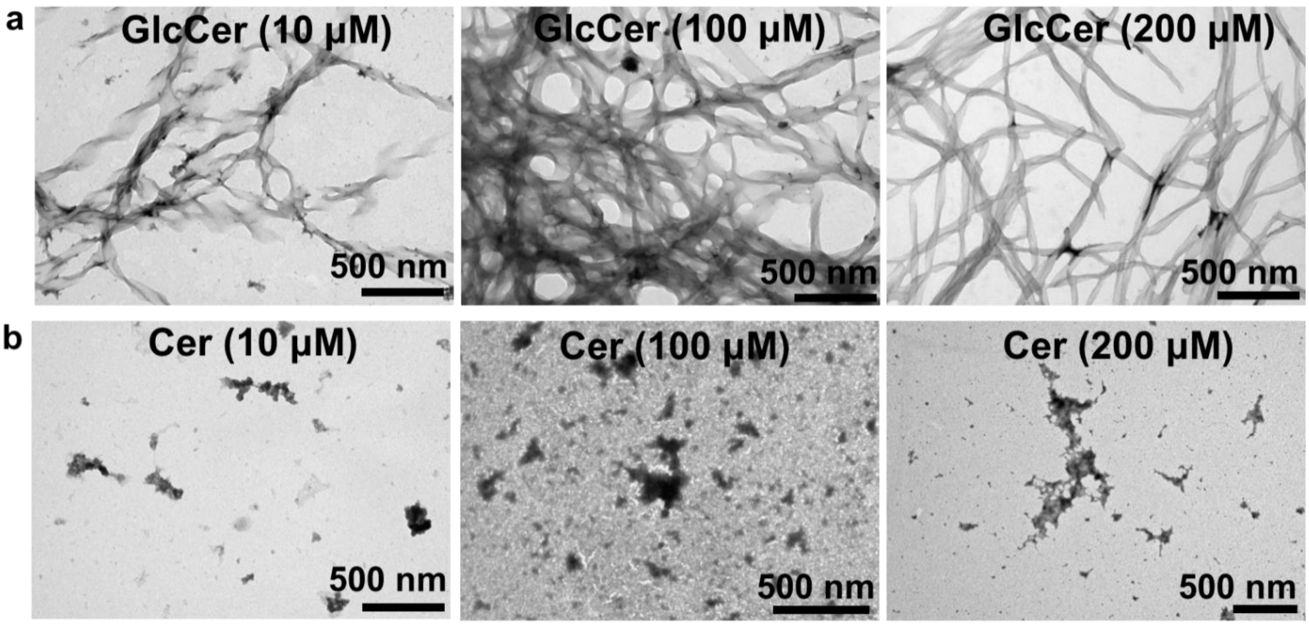

Figure S22. Morphology of GlcCer and Cer assemblies. TEM images of different concentrations of (a) GlcCer and (b) Cer samples incubated in AB pH 4.5 at 37ºC for 10 h.
